# Small‐sized mesenchymal stem cells with high glutathione dynamics show improved therapeutic potency in graft‐versus‐host disease

**DOI:** 10.1002/ctm2.476

**Published:** 2021-07-04

**Authors:** Jisun Lim, Jinbeom Heo, Hwan Yeul Yu, HongDuck Yun, Seungun Lee, Hyein Ju, Yun Ji Nam, Seon Min Jeong, Jinwon Lee, You Sook Cho, Myung‐Soo Choo, Eui Man Jeong, Chae‐Min Ryu, Dong‐Myung Shin

**Affiliations:** ^1^ Department of Biomedical Sciences Asan Medical Center University of Ulsan College of Medicine Seoul Korea; ^2^ Department of Physiology Asan Medical Center University of Ulsan College of Medicine Seoul Korea; ^3^ Department of Urology Asan Medical Center University of Ulsan College of Medicine Seoul Korea; ^4^ Division of Allergy and Clinical Immunology Department of Internal Medicine Asan Medical Center University of Ulsan College of Medicine Seoul Korea; ^5^ Jeju Research Institute of Pharmaceutical Sciences College of Pharmacy Jeju National University Jeju Korea; ^6^ Interdisciplinary Graduate Program in Advanced Convergence Technology and Science Bio‐Health Materials Core‐Facility Center and Practical Translational Research Center Jeju National University Jeju Korea

Dear Editor,

Graft‐versus‐host disease (GVHD) is a major complication of allogeneic hematopoietic stem cell (SC) transplantation with standard first‐line treatment consisting of administration of systemic high‐dose steroids.^1^ Unfortunately, effective therapies and standards of care are lacking for second‐line treatment in patients with steroid‐refractory disease, resulting in poor prognosis.^1^ Mesenchymal‐SCs (MSCs) have regenerative, immunomodulatory, and anti‐inflammatory properties,^2^ making their administration a promising strategy to treat incurable GVHD.^2^, ^3^ MSCs, however, are heterogeneous, with differences in their morphologic and molecular characteristics, leading to different outcomes in preclinical and clinical studies.^4^

We previously demonstrated that small‐sized MSCs enriched by hypoxic conditions represent the most primitive population of MSCs, showing enhanced stemness and immunomodulatory effects.^5^ In addition, glutathione (GSH) dynamics, which represent cellular antioxidant capacity, are key in determining the core functions and therapeutic efficacy of human MSCs through a signaling cascade involving cyclic adenosine monophosphate response element–binding protein‐1 (CREB1) and nuclear factor (erythroid‐derived‐2) like‐2 (NRF2).^6^, ^7^ Primitive MSCs have exhibited therapeutic efficacy in cell culture‐based assays and a humanized GVHD mouse model.^5^, ^7^

Primitive MSCs, which reside in specific niches *in vivo*, are extremely difficult to stabilize *in vitro*, largely due to chronic oxidative stress and epigenetic instability.^8^ To overcome this drawback, MSCs were treated with ascorbic acid 2‐glucoside (AA2G), a stable vitamin‐C derivative that enhances the primitiveness and epigenetic integrity of MSCs through a CREB1‐dependent mechanism.^9^ Indeed, human umbilical‐cord derived MSCs (UC‐MSCs) cultured with 0.74 mM AA2G stimulated the translocation of NRF2 protein into the nucleus and upregulated the expression of major genes targeted by CREB1‐NRF2 (e.g., *GCLM*, *GCLC*, *PRDX1*, and *GSR*), indicating that the NRF2 pathway was activated and redox homeostasis preserved in MSCs^7^ (Figure S1). Further stimulation of these AA2G‐primed MSCs with sphingosine‐1‐phosphate (S1P) and valproic acid (VPA) improved their *in vivo* engraftment.^10^ This novel *ex vivo* expansion procedure was termed Primed/Fresh/OCT4 (PFO) enrichment.

We first examined whether altering the duration of AA2G treatment could affect the maintenance of primitive small‐sized MSCs (Figure S2A). Flow cytometric and microscopic analyses showed that populations of enlarged cells were progressively increased as UC‐MSCs were expanded under normal (naïve) culture conditions. The PFO‐procedure, however, protected UC‐MSCs (PFO/UC‐MSCs) from replication‐induced cell size enlargement (Figures 1A and 1B). This protective effect was first observed after 4 days of AA2G supplementation and was sustained over eight passages (11 days) of expansion of UC‐MSCs from two independent donors (Figures S2B–S2D), with little change in their multi‐potency and cell surface phenotypes (Figure S3). The PFO‐procedure was also effective for preserving small‐sized adipose‐derived MSCs (AD‐MSCs) (Figure S4).

**FIGURE 1:**
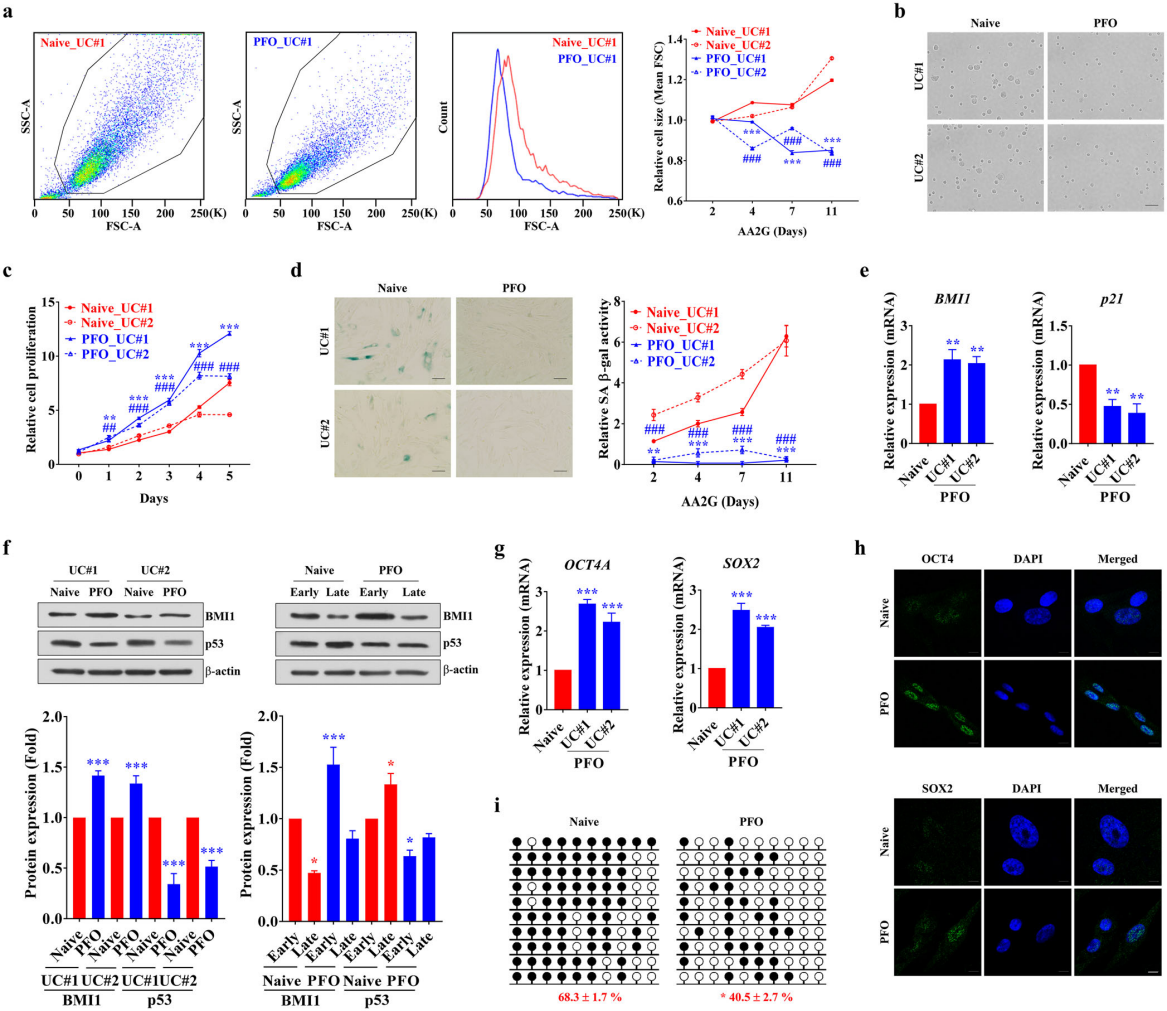
*In vitro* stabilization of primitive small‐sized UC‐MSCs by the PFO‐procedure. (A and B) Flow cytometric (A) and microscopic (B) analyses of the sizes of normal (naïve) human UC‐MSCs and of UC‐MSCs subjected to the PFO‐procedure by treatment with AA2G for the indicated number of days. Quantitative results are presented as ratios of the sizes of PFO‐ to naïve UC‐MSCs, with the latter set at 1. UC‐MSCs from two independent donors (#1 and #2) were used (*n *= 5 for each donor MSC). **p < 0.01, ***p < 0.001 compared with UC#1; ##*p* < 0.01, ###*p* < 0.001 compared with UC#2. Representative flow cytometry results (A) and microscopic images in suspension (B, × 200 magnification, scale bar = 100 μm) show PFO/UC‐MSCs treated with AA2G for 7 days. (C and D) Growth kinetics (C, *n *= 4) and SA β‐gal staining (D, *n *= 7) assays during *ex vivo* expansion for 11 days of naïve and PFO UC‐MSCs. Cell proliferation was determined by MTT assays. (D) Representative images (AA2G treatment for 7 days) are shown at × 200 magnification. Scale bar = 100 μm. (E) Quantitative results (*n *= 4) of transcript levels of genes associated with replicative senescence (*p21*) and stemness (*BMI1)*. (F) Western blotting (upper panel) and quantification (lower panel, *n *= 4) of BMI1 and p53 proteins in naïve and PFO UC‐MSCs from two independent donors (UC#1 and UC#2) treated with AA2G for 7 days (left panel) and in early and late passage samples (right panel). The levels of expression of BMI1 and p53 proteins were normalized relative to the levels of the loading control, β‐actin, in the same samples. (G and H) Quantitative results (*n *= 4) of transcript levels of *OCT4* and *SOX2* genes (G) and immunofluorescence staining of OCT4 and SOX2 proteins (h) (green, × 1000 magnification, scale bar = 10 μm) in naïve and PFO UC‐MSCs. Nuclei were counterstained with DAPI (blue). (I) Bisulfite sequencing results (*n *= 4) of the human *OCT4* promoter in naïve and PFO UC‐MSCs. Methylated and unmethylated CpG sites in bisulfite sequences are shown as filled and open circles, respectively. The percentage of methylated CpG sites is shown under each BSS result profile. All quantitative data are shown as the means ± SEM. Statistical analyses were performed using one‐way (E and G) or two‐way (A, C, and F) ANOVA with Bonferroni *post hoc* tests or non‐parametric Mann–Whitney U tests (I). *p < 0.05, **p < 0.01, ***p < 0.001 compared with naïve UC‐MSCs

Small‐sized UC‐MSCs enriched by the PFO‐procedure were resistant to replicative senescence, with enhanced proliferation activity (Figures 1C, S5A, and S5B) and little expression of senescence‐associated β‐galactosidase (Figures 1D and S5C). PFO/UC‐MSCs exhibited lower expression of cyclin‐dependent kinase inhibitors, such as p21^CIP1^ and p53, but higher expression of the stemness marker BMI1, than naïve cells (Figures 1E and 1F). PFO/UC‐MSCs consistently expressed the pluripotency‐specific transcripts *OCT4A* and *SOX2* (Figure 1G), findings validated by the intra‐nuclear staining of OCT4 and SOX2 proteins (Figure 1H). In addition, the *OCT4* promoter of PFO/UC‐MSCs had an open chromatin structure with lower levels of DNA methylation (Figure 1I). Moreover, these cells were enriched in transcriptionally favorable histone modifications (Figure S6).

To assess whether the PFO‐procedure could affect the GSH dynamics of MSCs, the qualitative and quantitative aspects of GSH‐recovering capacity (GRC) were monitored in real‐time (Figure 2A) using FreSHtracer, a reversible chemical probe for GSH that allows non‐destructive, integrated, and image‐based high‐throughput assays.^6^, ^7^ Compared with naïve cells, PFO/UC‐MSCs exhibited higher basal GSH levels and GRC activity after a short exposure to 100 μM diamide, a thiol‐specific oxidant (Figure 2B). In agreement with results showing that GRC activity was representative of the core functions of MSCs,^7^ PFO/UC‐MSCs exhibited higher colony forming unit‐fibroblast (CFU‐F) activity (Figure 2C), chemoattraction to platelet‐derived growth‐factor (PDGF) (Figure 2D), and anti‐inflammatory potency (Figures 2E and S7A) than naïve cells. The PFO‐procedure also had beneficial effects on AD‐MSCs derived from two independent donors (Figure S8). In addition, genes related to stemness, cell migration, growth‐factors, chemokines, inflammation, and immune‐modulation were upregulated in PFO/UC‐MSCs (Figures 2F and S9).

**FIGURE 2:**
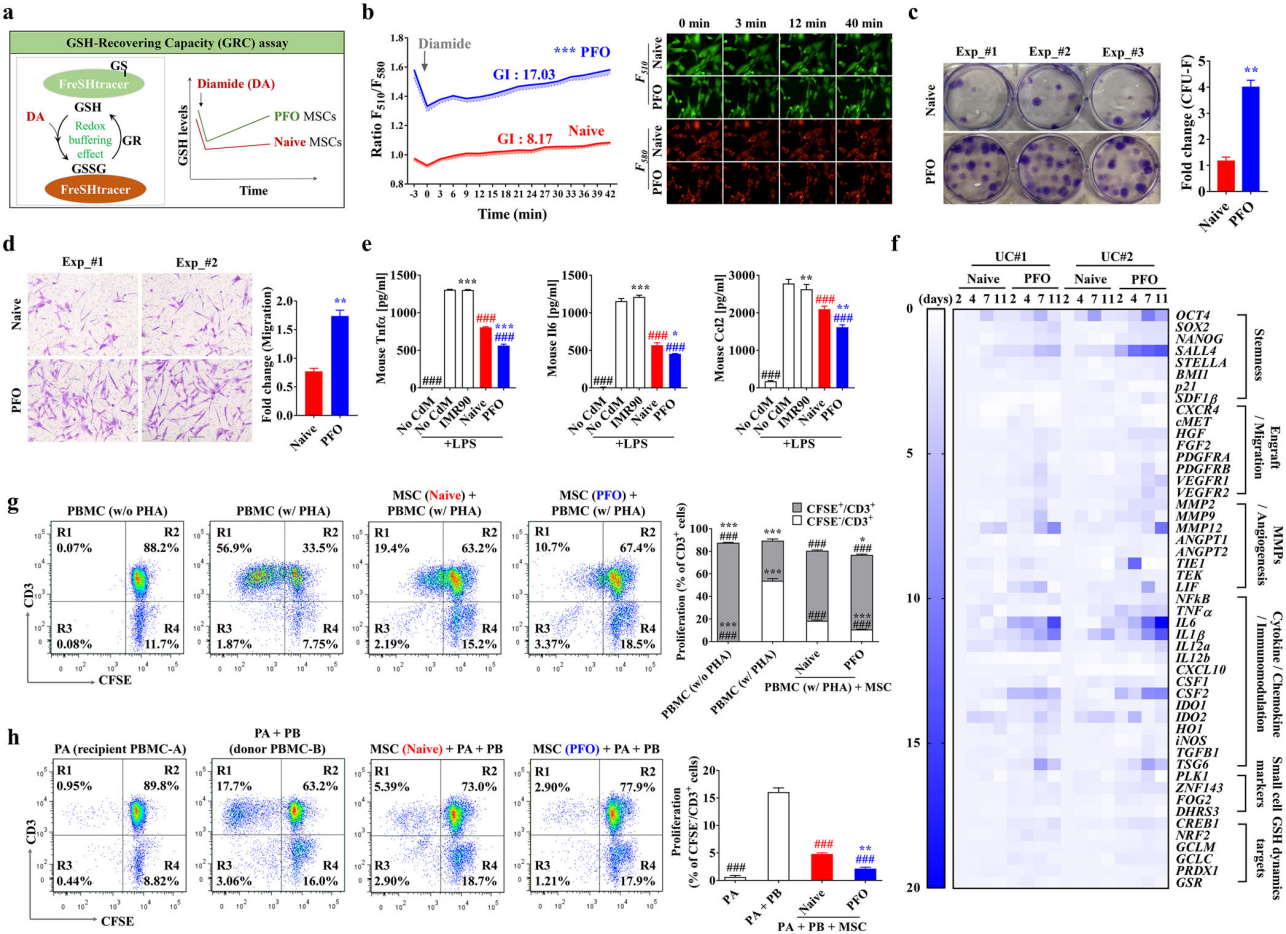
Enhanced immunomodulatory activities of small‐sized UC‐MSCs with high GSH dynamics. (A and B) Schematic overview (A) and FR plot (B) of the GSH‐recovering capacity (GRC) and basal GSH levels in naïve and PFO UC‐MSCs, generated by AA2G treatment for 7 days, as shown by FreSHtracer, in response to exposure to 100 μM diamide (arrow). The GSH dynamics index (GI) of each sample was quantified based on both initial F_510_/F_580_ fluorescence ratio (FR) (for baseline total GSH) and slope after diamide treatment (for GRC), as previously described.^7^ (B) FR plots with GI values (left panel) for each group (*n *= 6) and representative images (right panel) of F_510_ (GSH bound) and F_580_ (GSH free) fluorescence. (C and D) Colony forming unit‐fibroblast activity (C, CFU‐F) and chemotactic transwell migration activity in response to platelet‐derived growth‐factor (D, PDGF) of naïve and PFO UC‐MSCs generated by AA2G treatment for 7 days. Representative results of each assay are shown in the left panel (D, × 200 magnification; scale bar=100 μm). Data are presented as mean ± SEM (*n *= 6) ratios relative to naïve cells and analyzed by non‐parametric Mann–Whitney *U* tests. **p < 0.01 compared with the naïve MSC group. (E) *In vitro* anti‐inflammation assays using conditioned media (CdM) prepared from the indicated UC‐MSCs. Secretion of mouse pro‐inflammatory cytokines, including tumor necrosis factor‐α (Tnfα), interleukin‐6 (Il6), and C‐C motif chemokine ligand‐2 (Ccl2), by MH‐S cells, a murine alveolar macrophage cell line stimulated with LPS for 8 h in the absence or presence of CdM harvested from the indicated cells. IMR90, a normal primary fibroblast line, was used for control. These data are reported as the mean ± SEM (*n *= 4) ratios relative to naïve MSCs and analyzed by one‐way ANOVA with Bonferroni *post hoc* tests. *p < 0.05, **p < 0.01, ***p < 0.001 compared with naïve cells. ###*p* < 0.001 compared with LPS‐stimulated MH‐S in the absence of CdM (No CdM). (F) Heatmap analysis of RQ‐PCR assays showing the expression of the indicated genes in naïve and PFO human UC‐MSCs from two independent donors (UC#1 and UC#2) generated by AA2G treatment for the indicated number of days. Levels of expression are shown as fold changes relative to naïve MSCs. Source data are available in Dataset S1. (G and H) Representative flow cytometry cytograms and quantitative (*n *= 4) results of the suppression of T‐cell proliferation (CFSE^−^/CD3^+^) in PHA‐stimulated (G) and allogeneic stimulated (H) PBMCs by co‐culture with naïve and PFO UC‐MSCs (AA2G for 7 days). (H) In the mixed lymphocyte reaction assays, single (PA) and allogeneic PBMCs (PA + PB) were co‐cultured with the indicated human UC‐MSCs. Statistical analyses were performed using one‐way (H) or two‐way (G) ANOVA with Bonferroni *post hoc* tests. **p < 0.01, ***p < 0.001 compared with naïve MSCs. ###*p* < 0.001 compared with controls for each assay (G, PBMC treated with PHA alone; H, PA + PB)

Evaluation of immune‐modulation activity showed that PFO/UC‐MSCs suppressed *in vitro* proliferation of CD3^+^ T‐cells in human peripheral blood mononuclear cells (PBMCs) by stimulating phytohemagglutinin (Figure 2G). PFO/UC‐MSCs strongly inhibited the proliferation of PBMCs upon allogeneic stimulation (Figure 2H) and secreted higher levels of PGE2,^5^ a soluble immune‐modulatory factor, than naïve cells (Figure S7B). Collectively, these results demonstrate that the PFO‐procedure can enrich for small‐sized primitive MSCs with higher GSH dynamics that are resistant to senescence, while possessing the enhanced core functions of MSCs.

To confirm the *in vivo* relevance of these findings, the therapeutic potency of PFO/UC‐MSCs was investigated in a humanized GVHD mouse model induced by the transplantation of human PBMCs.^5^, ^7^ Although all mice transplanted with human PBMCs alone died within 60 days, those transplanted with naïve cultured and PFO‐processed UC‐MSCs had survival rates of 60% and 90%, respectively (Figure 3A). Weight loss was lower in GVHD mice transplanted with PFO/UC‐MSCs than with naïve cells (Figures 3B and S10). Injection of PFO/UC‐MSCs significantly ameliorated the clinical scoring and histological injuries in the organs targeted in GVHD, including the small intestine, lungs, liver, and kidneys (Figures 3C and 3D).

**FIGURE 3:**
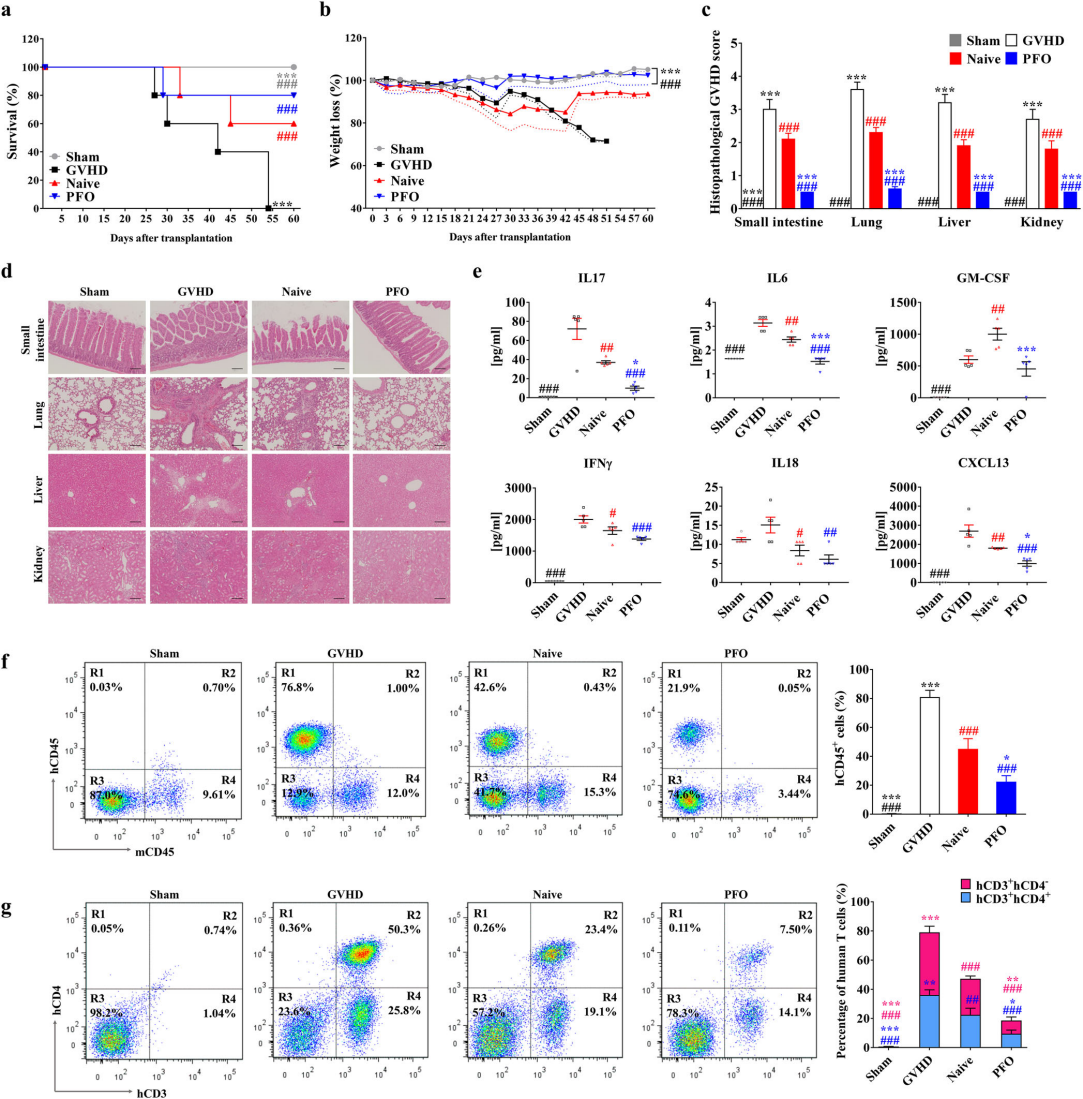
Enhanced therapeutic efficacy of PFO/UC‐MSCs in GVHD. (A and B) Survival rate (A) and body weight loss (B) evaluated daily for 60 days in mice with humanized GVHD (NOD.Cg‐*Prkdc^scid^Il2rg^tm1Wjl^*/SzJ), induced by intravenous injection of 1.0 × 10^6^ human PBMCs, followed by administration of 1 × 10^5^ human UC‐MSCs in normal (naïve) culture and subjected to the PFO‐procedure via the tail vein (*n *= 5 per group). As a sham control, PBS was injected instead of PBMCs. The dotted lines (B) depict the SEM. (C−G) Histological and immunological changes in the GVHD mice 6 weeks after infusion of human PBMCs (*n *= 5 per group). (C and D) Quantification (*n *= 10) of histological disease scores (C) and representative results of hematoxylin and eosin staining (D) (× 200 magnification; scale bar = 100 μm) of the indicated GVHD target organs. (E) Quantitative multiplex analysis of 28 human cytokines and chemokines in sera from the indicated GVHD mice (*n *= 5 per group). Results for other human cytokines are presented in Figure S11. (F and G) Suppression of donor T‐cell population in GVHD mice administered with human PFO/UC‐MSCs. Representative flow cytometric analysis of human (H) and mouse (m) CD45^+^ cells (F) and human T‐cells expressing CD3 (hCD3) or CD4 (hCD4) (G) in splenocytes of GVHD mice from the indicated groups. All quantitative data are presented as mean ± SEM (*n*=5). Statistical analyses were performed using one‐way (E and F) or two‐way (A−C, and G) ANOVA with Bonferroni post‐test. *p < 0.05, **p < 0.01, ***p < 0.001 compared with the naïve group. ##*p* < 0.01, ###*p* < 0.001 compared with the GVHD group

Mechanistic insight into these findings was provided by multiplex cytokine profiling of serum and flow cytometric analysis of splenocytes in GVHD mice 42 days after transplantation of human PBMCs. PFO/UC‐MSCs effectively reduced the serum concentrations of human cytokines and chemokines related to Th17 (e.g., IL17, IL6, and GM‐CSF) and Th1 (e.g., IFNγ and IL18) helper T‐cells and to pro‐inflammatory responses (e.g., CXCL13, TNFα, IL8, and IL23) (Figures 3E and S11). PFO/UC‐MSCs also further reduced the populations of human CD45^+^, CD3^+^, and CD3^+^CD4^+^ cells in the spleens of GVHD mice (Figures 3F and 3G), indicating that these cells have enhanced *in vivo* anti‐inflammatory and immunomodulatory activities.

The present study showed that the combination of three small molecules, AA2G, S1P, and VPA, provided an optimal environment for *in vitro* capture of primitive MSCs, small‐sized cells with high antioxidant capacity and therapeutic efficacy for treating GVHD (Figure S12). This simple procedure is potentially applicable for evaluating the functionality and/or therapeutic potency of other cell‐based therapies, including (i) MSCs derived from other sources,^5^ (ii) other types of SCs, (iii) OCT4‐expressing adult SCs, and (iv) immunomodulatory therapeutic cells such as Tregs, NKs, and NKTs. The significance and limitations of this study are discussed in detail in the Supplementary Notes.

## CONFLICT OF INTEREST

Dong‐Myung Shin is a cofounder of Cell2in, a company focused on developing FreSHtracer‐based assays. The other authors have no conflict of interest to declare.

## AUTHOR CONTRIBUTIONS

*Conceptualization*: Dong‐Myung Shin, Jisun Lim, Jinbeom Heo, Hwan Yeul Yu, and Chae‐Min Ryu. *Methodology*: Dong‐Myung Shin, Jisun Lim, Jinbeom Heo, Chae‐Min Ryu, Yun Ji Nam, Seon Min Jeong, Jinwon Lee, You Sook Cho, Myung‐Soo Choo, and Eui Man Jeong. *Investigation*: Jisun Lim, Jinbeom Heo, Chae‐Min Ryu, HongDuck Yun, Seungun Lee, Hyein Ju, Hwan Yeul Yu, and Dong‐Myung Shin. *Writing of the original draft*: Dong‐Myung Shin and Jisun Lim. *Review and editing of the manuscript*: Dong‐Myung Shin, Jisun Lim, Jinbeom Heo, and Chae‐Min Ryu. *Acquisition of funding*: Dong‐Myung Shin, Jisun Lim, Jinbeom Heo, and You Sook Cho. *Resources*: Eui Man Jeong and You Sook Cho. *Data curation*: Dong‐Myung Shin, Jisun Lim, Jinbeom Heo, Hwan Yeul Yu, and Chae‐Min Ryu. *Supervision*: Dong‐Myung Shin.

## Supporting information

Figures S1‐S12

Supporting Information

## References

[1] Im A , Hakim FT , Pavletic SZ . Novel targets in the treatment of chronic graft‐versus‐host disease. Leukemia. 2017;31:543–554. 27899803 10.1038/leu.2016.367

[2] Blazar BR , MacDonald KPA , Hill GR . Immune regulatory cell infusion for graft‐versus‐host disease prevention and therapy. Blood. 2018;131:2651–2660. 29728401 10.1182/blood-2017-11-785865 PMC6032895

[3] Gao L , Zhang Y , Hu B , et al. Phase II multicenter, randomized, double‐blind controlled study of efficacy and safety of umbilical cord‐derived mesenchymal stromal cells in the prophylaxis of chronic graft‐versus‐host disease after HLA‐haploidentical stem‐cell transplantation. J Clin Oncol. 2016;34:2843–2850. 27400949 10.1200/JCO.2015.65.3642

[4] Galipeau J , Sensebe L . Mesenchymal stromal cells: clinical challenges and therapeutic opportunities. Cell Stem Cell. 2018;22:824–833. 29859173 10.1016/j.stem.2018.05.004 PMC6434696

[5] Kim Y , Jin HJ , Heo J , et al. Small hypoxia‐primed mesenchymal stem cells attenuate graft‐versus‐host disease. Leukemia. 2018;32:2672–2684. 29789652 10.1038/s41375-018-0151-8 PMC6286327

[6] Jeong EM , Yoon JH , Lim J , et al. Real‐time monitoring of glutathione in living cells reveals that high glutathione levels are required to maintain stem cell function. Stem Cell Reports. 2018;10:600–614. 29307581 10.1016/j.stemcr.2017.12.007 PMC5830891

[7] Lim J , Heo J , Ju H , et al. Glutathione dynamics determine the therapeutic efficacy of mesenchymal stem cells for graft‐versus‐host disease via CREB1‐NRF2 pathway. Sci Adv. 2020;6:eaba1334. 32490200 10.1126/sciadv.aba1334 PMC7239701

[8] Heo J , Lim J , Lee S , et al. Sirt1 regulates DNA methylation and differentiation potential of embryonic stem cells by antagonizing Dnmt3l. Cell Rep. 2017;18:1930–1945. 28228259 10.1016/j.celrep.2017.01.074

[9] Lee S , Lim J , Lee JH , et al. Ascorbic acid 2‐glucoside stably promotes the primitiveness of embryonic and mesenchymal stem cells through ten‐eleven translocation‐ and cAMP‐responsive element‐binding protein‐1‐dependent mechanisms. Antioxid Redox Signal. 2020;32:35–59. 31656084 10.1089/ars.2019.7743

[10] Lim J , Lee S , Ju H , et al. Valproic acid enforces the priming effect of sphingosine‐1 phosphate on human mesenchymal stem cells. Int J Mol Med. 2017;40:739–747. 28677769 10.3892/ijmm.2017.3053 PMC5547989

