# Supplementary material for: Small‐sized mesenchymal stem cells with high glutathione dynamics show improved therapeutic potency in graft‐versus‐host disease
Source: Clin Transl Med. 2021 Jul 4;11(7):e476. doi: 10.1002/ctm2.476 (PMC8255063; doi:10.1002/ctm2.476)
Supplement: Supplementary file 1 — Figures S1‐S12 [file CTM2-11-e476-s001.docx]

**Supplementary Information**

**Small-sized mesenchymal stem cells with high glutathione dynamics show improved therapeutic potency in graft-versus-host disease.**

Jisun Lim^†^, Jinbeom Heo^†^, Hwan Yeul Yu, HongDuck Yun, Seungun Lee, Hyein Ju, Yun Ji Nam, Seon Min Jeong, Jinwon Lee, You Sook Cho, Myung-Soo Choo, Eui Man Jeong, Chae-Min Ryu^*^, Dong-Myung Shin^*^

^†^These authors contributed equally to this work.

*Corresponding authors. [d0shin03@amc.seoul.kr](mailto:d0shin03@amc.seoul.kr) and chaemin0427@ulsan.ac.kr

**This PDF file includes:**

Supplementary Methods

Figures S1 to S13 and figure legends

Supplementary Notes

Table S1 (Key resource table)

Captions for Dataset S1 and S2

Supplementary References

Uncropped western blot results

**Other Supplementary Information include separate files for**

**Dataset S1.** Source data for quantification analyses

**Dataset S2.** Values for the GI index for each plot

**SUPPLEMENTARY METHODS**

***Study approval***

Human umbilical cord (UC) and abdominal adipose samples were obtained from healthy, normal, full-term newborns, and pregnant mothers, respectively, after obtaining written informed consent from the latter. All procedures were performed in accordance with the guidelines approved by the Ethics Committee on the Use of Human Subjects at Asan Medical Center (IRB#: 2015-0303). All animal experiments were approved by the Institutional Animal Care and Use Committee of the University of Ulsan College of Medicine (IACUC-2020-12-141).

***Cell culture of mesenchymal stem cells (MSCs)***

Human UCs were collected and MSCs (UC-MSCs) were isolated as previously described.^1^ Human UC-MSCs were grown in low-glucose DMEM containing 10% heat-inactivated fetal bovine serum (FBS; HyClone, Pittsburgh, PA), 5 ng/mL human epidermal growth factor (EGF; Sigma-Aldrich, St. Louis, MO, USA), 10 ng/mL basic fibroblast growth factor (bFGF), and 50 ng/mL long-R3 insulin-like growth factor-1 (IGF-1; ProSpec, Rehovot, Israel), as described.^2-4^ Human adipose tissue derived MSCs (AD-MSCs), established as described,^5^ were maintained using the same procedure as for UC-MSCs. All MSCs used in this study were expanded for fewer than seven passages to ensure their functionality and were maintained at 37°C in a humidified atmosphere containing 5% CO_2_.

For the PFO procedure, MSCs were plated at a density of 7 × 10^4^ cells/mL and maintained in culture medium supplemented with 0.74 mM ascorbic acid 2-glucoside (AA2G; Sigma-Aldrich) for the indicated number of days. One day before functional evaluation, 50 nM sphingosine 1-phosphate (S1P; Sigma-Aldrich) and 0.5 mM valproic acid (VPA; Sigma-Aldrich) were added to the culture medium containing 0.74 mM AA2G to improve *in vivo* engraftment of MSCs, as previously described.^2^

***In vitro cell proliferation, self-renewal, multipotency, and migration capacities of MSCs***

*In vitro* cellular activities of MSCs, including colony forming unit-fibroblast (CFU) activity for self-renewal, multipotency (*in vitro* differentiation into chondrogenic, osteogenic, or adipogenic lineages), and trans-well migration in response to platelet-derived growth factor (PDGF; 10 ng/mL PDGF-AA, R&D Systems, Minneapolis, MN, USA) were analyzed as previously described.^3,4,6-8^ These core functions of MSCs were quantified by digital image analysis using Image Pro 5.0 software (Media-Cybernetics, Rockville, MD, USA). Cell proliferation capacity was determined by MTTs assay (Sigma-Aldrich) and further validated by bromodeoxyuridine (BrdU) incorporation enzyme-linked immunosorbent assay (ELISA) kits (#11647229001, Roche, Mannheim, Germany).

***In vitro anti-inflammation and immune modulation activities of MSCs***

*In vitro* anti-inflammation, immune modulation, and inhibitory properties in response to stimulation by allogeneic mixed lymphocyte reactions (MLRs) were assessed as previously described.^3,4,6-8^

For *in vitro* assays of anti-inflammatory activity, MH-S, a murine alveolar macrophage cell-line (ATCC, Manassas, VA, USA) was cultured in high-glucose DMEM (Hyclone) supplemented with 10% heat-inactivated FBS and penicillin/streptomycin. Each well of a 12-well culture plate was seeded with 7.5 × 10^5^ MH-S cells, followed by activation with lipopolysaccharide (LPS) (0.1 μg/mL; Sigma-Aldrich). Conditioned media (CdM) were harvested from UC-MSCs or IMR90 after naïve culture or subjection to the PFO procedure for 2 days. The LPS-stimulated MH-S cells were co-cultured with the CdM for 8 hours, and the concentrations of mouse tumor necrosis factor-α (Tnfα), interleukin-6 (Il6), Il1β, and C-C motif chemokine ligand-2 (Ccl2) were measured in the culture media using ELISA kits as described in **Supplementary Table S1**. The concentrations of prostaglandin E2 (PGE2), a soluble factor responsible for the immunoregulatory effects of MSCs,^9^ were measured in the CdM harvested from naïve culture or PFO UC-MSCs using Human Prostaglandin E2 ELISA Kits (#KHL1701, Invitrogen/Thermo Fisher Scientific, Waltham, MA, USA).

Allogenic MLR assays utilized human PBMCs derived from two different donors (#70025; STEMCELL Technologies, Vancouver, BC, Canada). Stimulator human PBMCs (donor PBMC-B; PB) were inactivated by irradiation at 5.0 Gy using the X-RAD 320 X-ray irradiator (Precision X-Ray, Inc., North Branford, CT, USA). Responder PBMCs (recipient PBMC-A; PA) and the inactivated donor PB were labeled by the CFSE. CFSE-labeled inactivated PB cells (2 × 10^5^) were added to each well of a 96-well culture plate containing human MSCs (2 × 10^4^) and the CFSE-labeled responder PA (1 × 10^5^). The cell mixtures were further incubated for 6 days at 37 °C in a humidified atmosphere containing 5% CO_2_. Cell proliferation activity was analyzed using the CFSE Cell Division Tracker Kit (#423801; BioLegend, San Diego, CA, USA), and the percentage of CD3^+^ T-cells (#300412; BD Biosciences, Mountain View, CA, USA) was analyzed using the BD FACS Canto II flow cytometer (BD Biosciences). FACS data were analyzed using FlowJo software 7.6.5 (FlowJo, LLC, Ashland, OR, USA).

***Flow cytometric analysis of MSCs***

To assess cell surface protein expression, 1.0 × 10^5^ MSCs were resuspended in high-glucose DMEM containing 2% FBS and incubated with the indicated antibodies for 30 min on ice, washed twice, and analyzed using a BD FACS Canto II flow cytometer (BD Biosciences). Cells were incubated with the following fluorophore conjugated antibodies characteristic of MSCs,^9^ which were purchased from BD Biosciences or Invitrogen: FITC-conjugated, anti-CD14 (clone M5E2), PE-conjugated anti-CD29 (clone MAR4), PE-conjugated anti-CD34 (clone 581), PE-conjugated anti-CD45 (clone HI30), FITC-conjugated anti-CD49f (clone GoH3), PE-conjugated anti-CD73 (clone AD2), PE-conjugated anti-CD90 (clone 5E10), PerCP-Cy^TM^5.5-conjugated anti-CD105 (clone 266), and eFlour660-conjugated anti-S1PR1 (clone SW4GYPP).

***Humanized graft-versus-host disease (GVHD) animal model***

Nine-week-old male NOD.Cg-*Prkdc^scid^Il2rg^tm1Wjl^*/SzJ (NSG) mice weighing 26–29 g, purchased from Jackson Laboratory (JAX, Bar Harbor, ME, USA), were irradiated at 2.0 Gy using an X-RAD 320 X-ray irradiator (Precision X-Ray, Inc), as described.^4,6,10^ Within 24 hours, the mice were injected via the tail vein with 1.0 × 10^6^ human PBMCs (#70025; STEMCELL Technologies) to induce GVHD or with the same volume of phosphate-buffered saline (PBS; Sham group). Eighteen days later, the mice were injected via the tail vein with 1 × 10^5^ human UC-MSCs expanded under normal (naïve) culture conditions or using the PFO procedure in 100 μL PBS; Sham and GVHD mice were injected with PBS alone as a control. The mice were randomly allocated to treatment groups (*n*=5 per group), and the order of irradiation, cell transplantation and vehicle injection, as well as daily examinations, were randomized. Investigators involved in GVHD induction procedures were blinded to treatment.

***Assessment of GVHD in animals***

Clinical symptoms of GVHD were evaluated daily by examining body weight loss, survival, hunched back, and fur texture, and were recorded every second day in five mice per group for 60 days after GVHD induction.^11^ To obtain mechanistic insights into MSC therapy, an independent set of five mice per group were used for histological assessment of GVHD target organs (lungs, liver, kidneys, and small intestine), immunological analyses of donor T cell populations, and multiplex human cytokine assays 6 weeks after administration of human PBMCs, as previously described.^4,6^ All GVHD symptoms and histological assessments, and all measurements of cytokines and splenocytes, were evaluated by blinded investigators.

To analyze donor T cell populations, cells isolated from the spleen of each GVHD mouse^12^ were resuspended in 100 μl PBS containing 2% FBS and incubated for 30 min at 4°C with APC-, PE/Cy7-, FITC-, or PE-conjugated primary antibodies against human antigens CD3 (#300412), CD4 (#300512), and CD45 (#555483), and mouse antigen CD45 (#553079), all purchased from BD Biosciences. The fluorescence intensity of cells was analyzed using the BD FACS Canto II flow cytometer (BD Biosciences), and the data were analyzed using FlowJo software 7.6.5 (FlowJo).

For multiplex cytokine analysis, sera from humanized GVHD mice were analyzed using the Magnetic Luminex Screening Assay human Premixed Multi-Analyte Kit (#LXSAHM-28; R&D Systems). The 28-plex human cytokines were measured and quantified by KOMA BIOTECH INC. (Seoul, Korea) using the Varioskan Flash Reader (Invitrogen), according to the manufacturer’s instructions.

***Real-time monitoring of GSH-recovering capacity (GRC) of living MSCs***

Real-time trace GSH changes in every living single cell under different culture conditions were assessed using the GRC assay and an Operetta High-Content Imaging System (HH12000000; PerkinElmer, Waltham, MA, USA) at ×200 or ×400 magnification, as described.^4^ This GRC assay allows non-destructive, integrated, and image-based high-throughput assays for qualitative and quantitative aspects of GSH dynamics in living MSCs. The GRC assay was based on the unique properties of FreSHtracer (**F**luorescent **re**al-time **thiol tracer**; Cell2in, Inc., Seoul, Korea), a reversible chemical probe for GSH.^7,13^ Upon reacting with GSH, FreSHtracer shows a spectral shift in the λ_max_ of its ultraviolet-visible absorption from 520 nm to 430 nm, resulting in decreased fluorescence emission intensity at 580 nm (F_580_, λ_ex_ 520 nm) and increased fluorescence intensity at 510 nm (F_510_, λ_ex_ 430 nm).^7,13^ Thus, to determine the fluorescence ratios (FR) of FreSHtracer, fluorescence emissions were measured at 510 and 580 nm after excitation at 430 and 520 nm, respectively. These fluorescence signals were analyzed using Harmony High-Content Imaging and Analysis Software 3.1 (PerkinElmer) in the confocal mode. The GSH dynamics indices (GI), related initial FRs (for baseline total GSH) and slopes after diamide treatment (for GSH recovery capacity [GRC]) of each plot are presented in **Supplementary Dataset 2**.

***Gene expression and western blotting analysis***

Transcripts of the indicated genes were quantified in 50 ng aliquots of total RNA, and their relative levels of expression were determined using the 2^-ΔΔCt^ method, with *GAPDH* mRNA as the endogenous control.^14,15^

For western blot analysis, cell extracts (30 μg) in RIPA lysis buffer (Santa Cruz Biotechnology, Santa Cruz, CA, USA) were electrophoresed on SDS-PAGE gels and transferred to nitrocellulose membranes, as described.^14,15^ The levels of expression of BMI1, p53, and β-actin were assessed by probing with specific antibodies (**Supplementary Table S1**). The density of signals for the indicated proteins was measured and quantified using NIH Image J software. The expression level of each indicated protein was normalized relative to that of β-actin.

***Chromatin-immunoprecipitation (ChIP) and DNA methylation assays***

ChIP analysis was performed using a Magna ChIP G Kit (Millipore, Billerica, MA, USA), according to the manufacturer’s instructions. Details of these procedures, including the use of 3 μg of ChIP grade antibody against acetylated histone-3 (H3Ac), histone-3 tri-methylated at lysine-4 (H3K4me3), and histone-3 tri-methylated at lysine-27 (H3K27me3), have been described.^14,15^ The DNA methylation status of human *OCT4* promoter and of the differentially methylated region of the *IGF2*−*H19* imprinted locus was assessed using bisulfite sequencing, as described.^14^ All the primers used in the ChIP and DNA methylation assay are described in **Supplementary Table S1**.

***Immunostaining***

Human MSCs were fixed with 4% paraformaldehyde (Sigma-Aldrich) for 30 min, stained with antibodies specific to OCT4 (#MAB4419; Millipore), SOX2 (#Ab92494; Abcam), and NRF2 (#Ab62352; Abcam), and subsequently incubated with Alexa 488-conjugated anti-mouse (#A11001) and anti-rabbit (#A11008) antibodies (Thermo Fisher Scientific, Waltham, MA, USA), respectively. Nuclei were counterstained with DAPI (Sigma-Aldrich). Images were acquired using a ZEISS LSM710 confocal microscope system (Carl Zeiss, Munich, Germany).

***Availability of data and materials***

Information about all reagents, resources, and oligonucleotides are described in **Supplementary Table S1**.

***Statistical analysis***

Data were statistically analyzed using non-parametric Mann–Whitney tests or one- or two-way ANOVA with Bonferroni *post-hoc* tests. All analyses were performed using GraphPad Prism 7.0 software (GraphPad Software, La Jolla, CA, USA), with *P* < 0.05 considered statistically significant.

**SUPPLEMENTARY FIGURES AND LEGENDS**

**
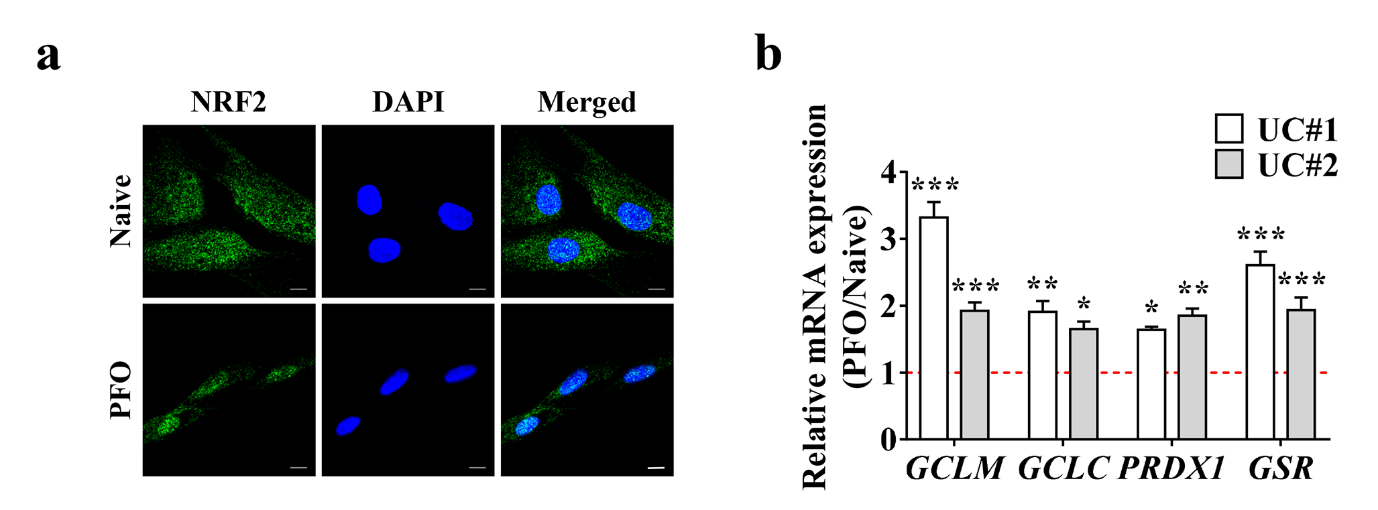
**

**Supplementary Fig. S1. Activation of the NRF2 pathway by the PFO procedure**

**(a)** Representative confocal microscopic images of NRF2 protein (green) in human UC-MSCs in normal (naïve) culture and after subjection to the PFO procedure, in which cells were cultured in the presence of AA2G for 7 days. Nuclei were stained with DAPI (blue), ×1,000 magnification. Scale bar=10 μm. Note that the PFO procedure induced the nuclear translocation of NRF2 protein. **(b)** Real-time quantitative PCR (RQ-PCR) assays of the expression of NRF2 dependent genes related to glutathione (GSH) synthesis (*GCLM* and *GCLC*) and redox cycling (*GSR* and *PRDX1*) in naïve and PFO UC-MSCs. Quantitative data are shown as the mean ± SEM (*n*=4) relative to the expression in naïve cultured cells (red dotted line). Statistical analyses were performed using two-way ANOVA with Bonferroni *post-hoc* tests. **P* < 0.05, ***P* < 0.01, ****P* < 0.001 compared with naïve MSCs. Abbreviations: *GCLM*, glutamate-cysteine ligase modifier subunit; *GCLC*, glutamate-cysteine ligase catalytic subunit; *GSR*, glutathione-disulfide reductase; *PRDX1*, peroxiredoxin 1.

**
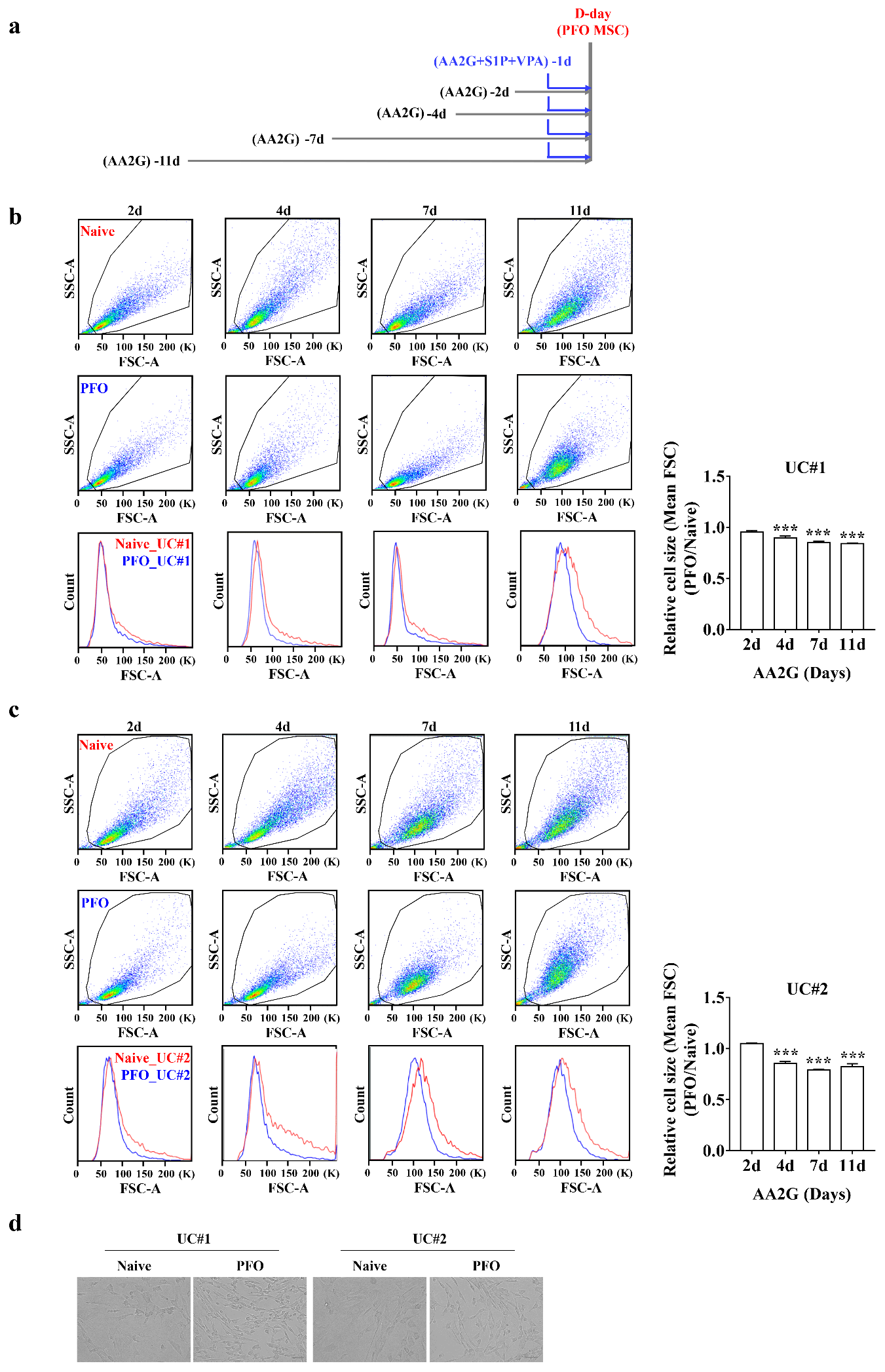
**

**Supplementary Fig. S2. Preservation of primitive small-sized UC-MSCs by the PFO procedure.**

**(a)** Schematic summary of the PFO procedure showing the numbers of days of AA2G supplementation. One day before functional evaluation, 50 nM sphingosine 1-phosphate (S1P) and 0.5 mM valproic acid (VPA) were added to the culture medium to improve *in vivo* engraftment of MSCs.^2^ **(b−d)** Sizes of human UC-MSCs from two independent donors (UC#1 and UC#2), as determined by **(b and c)** flow cytometry and **(d)** microscopy. **(b and c)** Quantification of flow cytometry results, with the sizes of UC-MSCs obtained with the PFO procedure (PFO UC-MSCs) shown relative to the sizes of naïve MSCs. Results are presented as means ± SEM (*n*=5 for each donor MSC) and compared by one-way ANOVA with Bonferroni *post-hoc* tests, ****P* < 0.001 compared with naïve cells. The optimal duration of AA2G supplementation was set at 7 days, based on the effects of AA2G on cell size and the expression of genes associated with the core functions of MSCs. The results of gene expression analyses are presented in detail in **Figure 2f and** **Supplementary Fig. 9**. **(d)** Representative images of naïve and PFO UC-MSCs (AA2G supplementation for 7 days) adhering to a tissue culture dish (×200 magnification). Scale bar=100 μm.


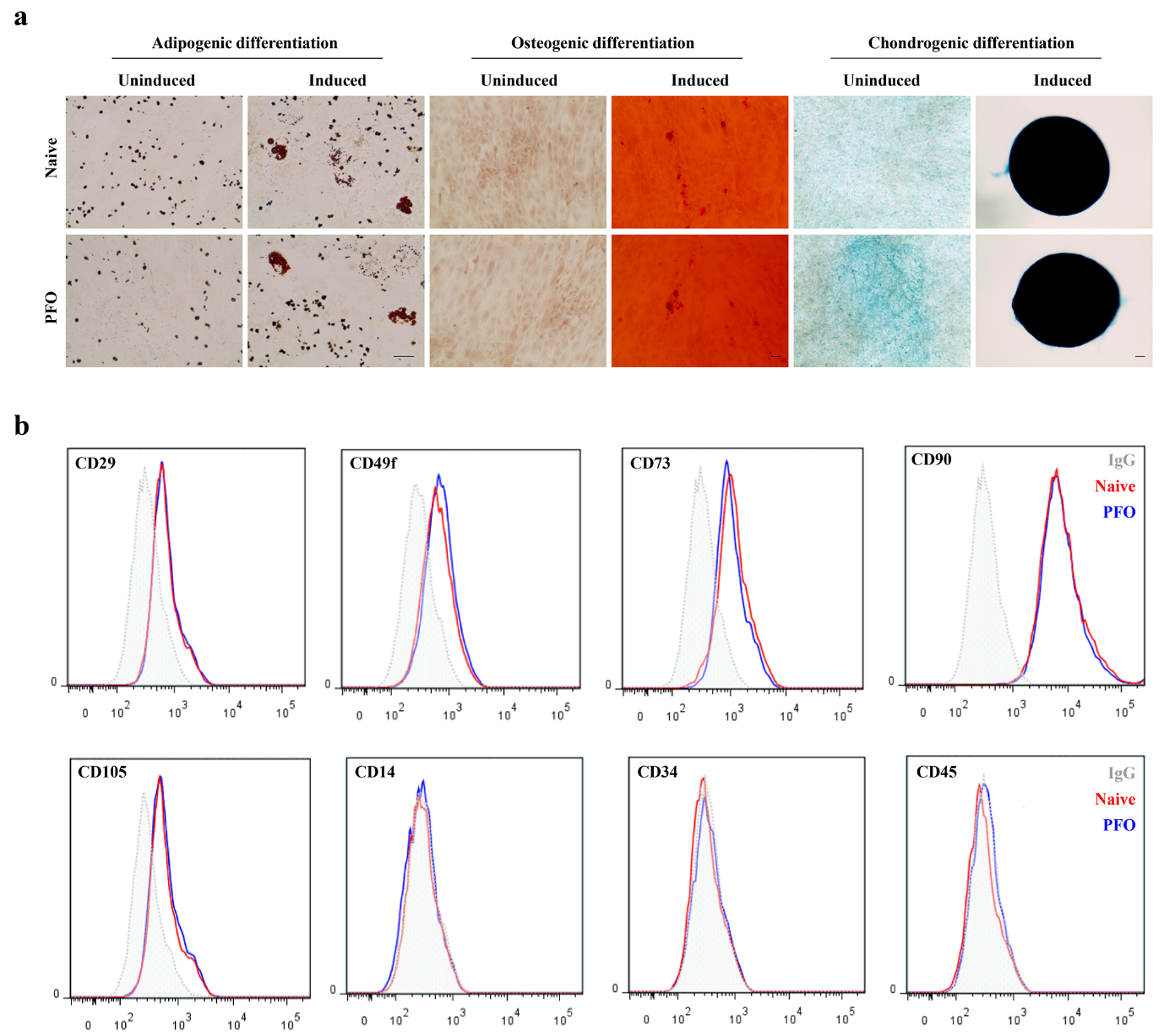


**Supplementary Fig. S3. Effect of the PFO procedure on the properties of MSCs**

**(a)** Multipotent differentiation of human UC-MSCs cultured in the absence (naïve) and presence (PFO procedure) of AA2G for 7 days on the adipogenic (left panel), osteogenic (middle panel), and chondrogenic (right panel) lineages, as assessed by staining with Oil Red O (×400 magnification), Alizarin Red S (×200 magnification), and Alcian Blue (×200 magnification), respectively (each scale bar=100 μm). **(b)** Flow cytometry analysis of the expression of surface proteins characteristic of MSCs (CD29, CD49f, CD73, CD90, and CD105) and hematopoietic cells (CD14, CD34, and CD45) on human naïve (red line) and PFO (blue line) UC-MSCs. Isotype IgG (gray dotted line) were used for negative control.

**
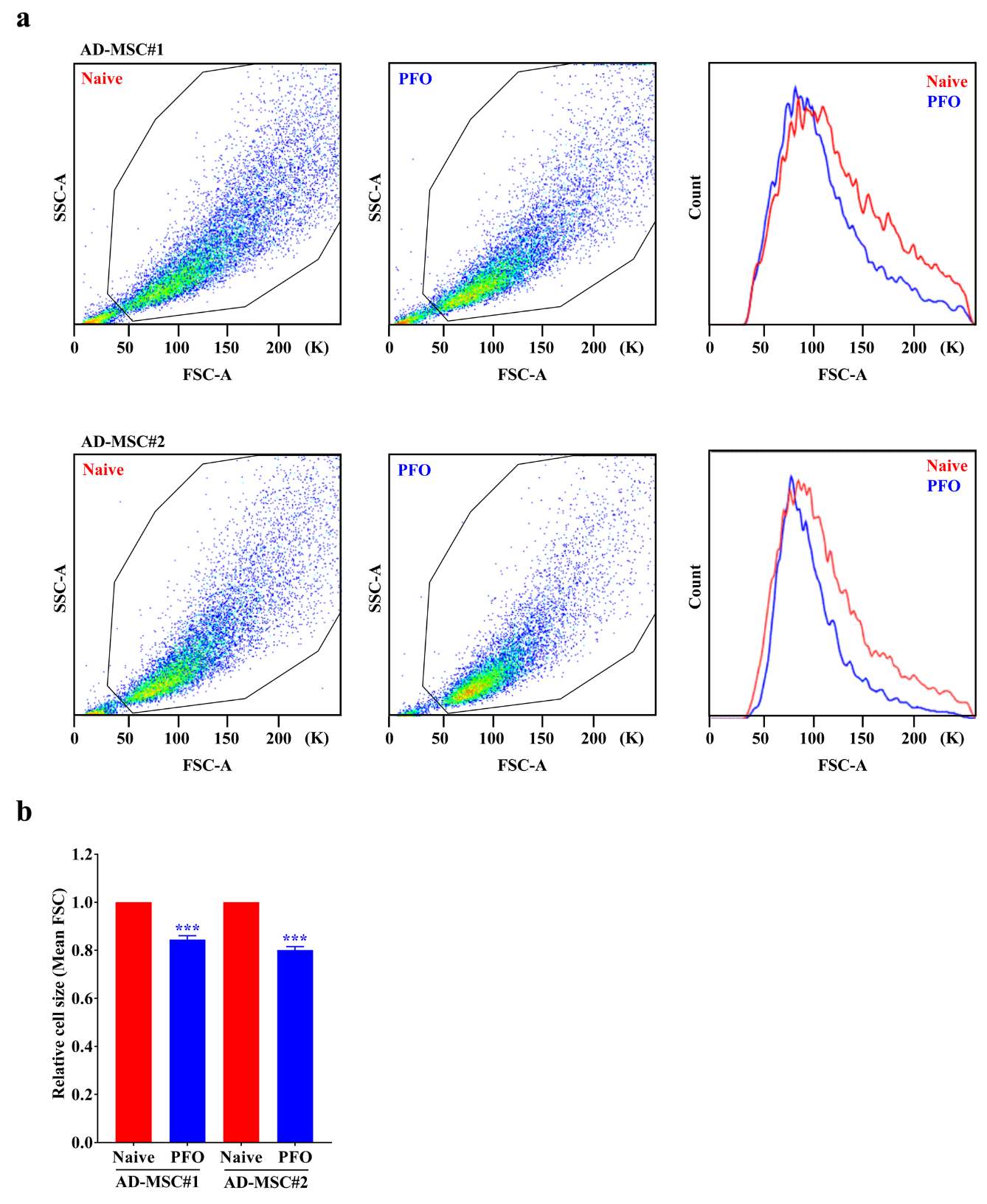
**

**Supplementary Fig. S4. Effect of the PFO procedure on human AD-MSCs**

**(a)** Representative flow cytometry cytograms measuring the sizes of human adipose derived-MSCs (AD-MSCs) from two independent donors (#1 and #2) after naïve culture or subjection to the PFO procedure (AA2G for 7 days). **(b)** Quantitatively determined ratios of the sizes of PFO treated to naïve MSCs. Results are presented as means ± SEM (*n*=4 for each donor MSC) and compared by one-way ANOVA with Bonferroni *post-hoc* tests, ****P* < 0.001 compared with naïve cells.


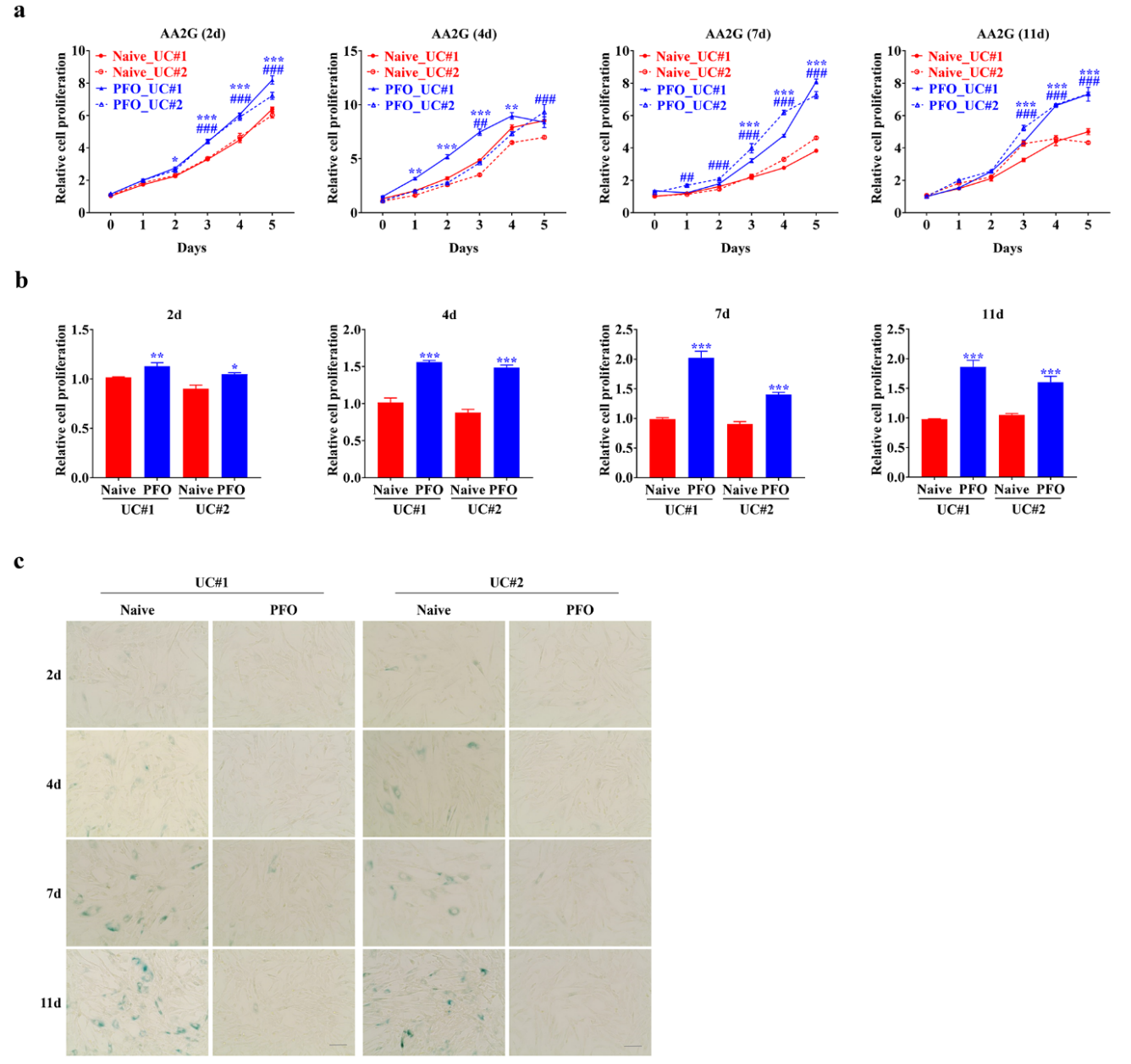


**Supplementary Fig. S5. PFO procedure protection of human UC-MSCs from replication induced senescence**

**(a and b)** Proliferation capacities of naïve and PFO UC-MSCs (from two independent donors), generated by AA2G treatment for 2, 4, 7, and 11 days, as determined by MTT assays **(a)** on the indicated days or bromodeoxyuridine (BrdU) incorporation activity **(b)** for 1 day. Quantitative data are shown as the mean ± SEM (*n*=4 for each donor MSCs) relative to naïve MSCs on day 1 and compared by one-way **(b)** or two-way **(a)** ANOVA with Bonferroni *post-hoc* tests, **P* < 0.05, ***P* < 0.01, ****P* < 0.001 compared with UC#1 **(a)** or naïve **(b)** cells, ##*P* < 0.01, ###*P* < 0.001 compared with UC#2 cells. **(c)** Representative images showing the expression of the senescence-associated marker β-galactosidase (SA β-gal) on naïve and PFO UC-MSCs from two independent donors (UC#1 and UC#2) (×200 magnification, scale bar=100 μm) as a function of the duration of AA2G supplementation. Quantitative determination of SA β-gal staining of the images shown in **Figure 1d**.


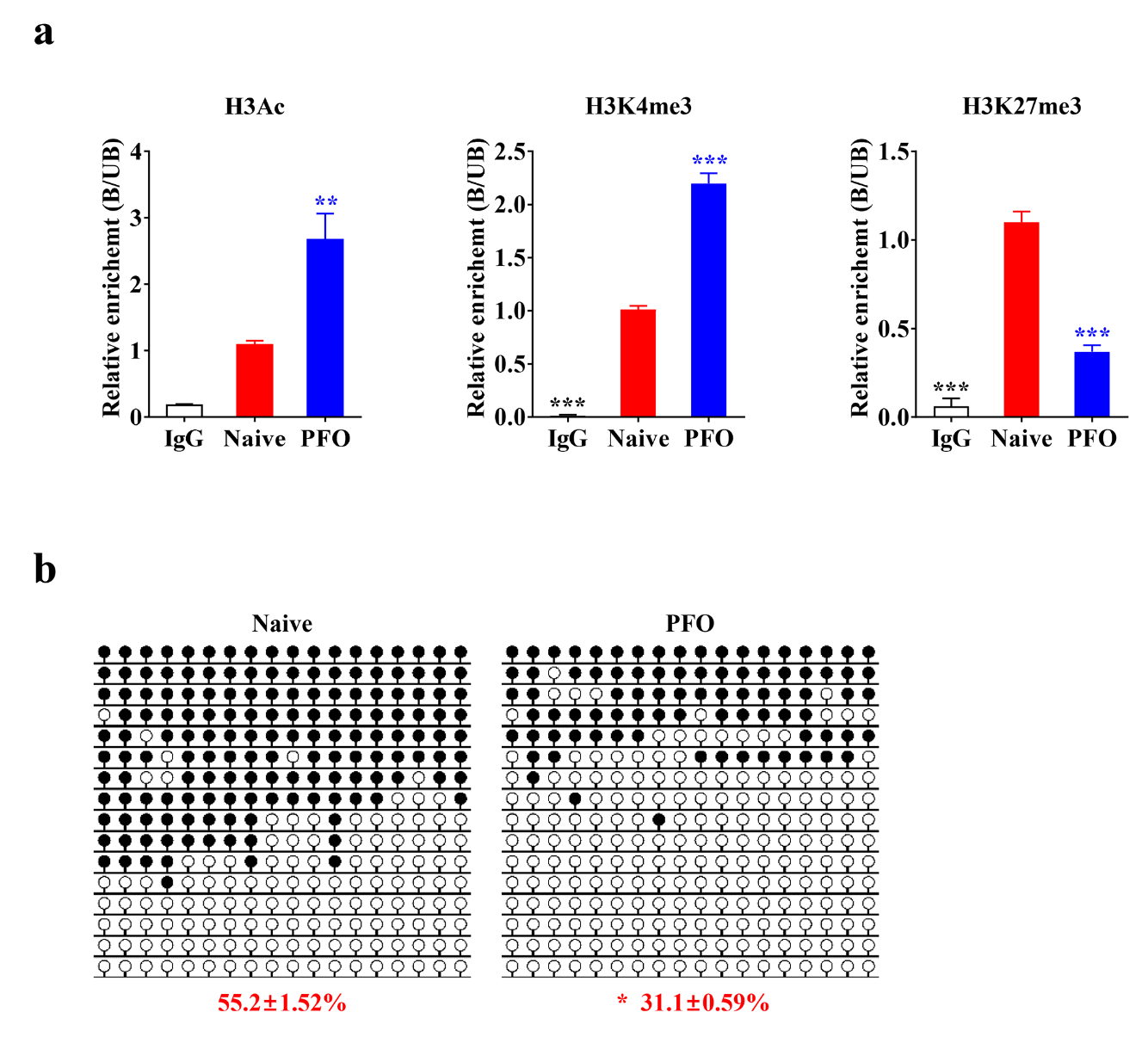


**Supplementary Fig. S6. Epigenetic status of *OCT4* promoter and *H19* imprint in PFO UC-MSCs**

**(a)** Quantitative chromatin immunoprecipitation (qChIP) analyses of acetylated histone H3 (H3Ac), H3 trimethylated at lysine 4 (H3K4me3), and H3 trimethylated at lysine 27 (H3K27me3) in naïve and PFO UC-MSCs. As controls, cells were incubated with isotype IgG. Enrichment of indicated histone modifications was calculated as the ratio of the bound (B) to the unbound (UB) fraction. Fold differences represent the ratio of PFO UC-MSCs to naïve MSCs. **(b)** Bisulfite sequencing of the differentially methylated region of *H19* paternally imprinted genes in the indicated cells. The numbers under each BSS profile indicate the percentage of methylated CpG sites. Methylated and unmethylated CpG sites in bisulfite sequences are shown as filled and open circles, respectively. All quantitative data are reported as means ± SEM (*n*=4). ***P* < 0.01, ****P* < 0.001 compared with the naïve group by one-way ANOVA with Bonferroni *post-hoc* tests **(a)** or non-parametric Mann–Whitney *U* tests **(b)**.

**
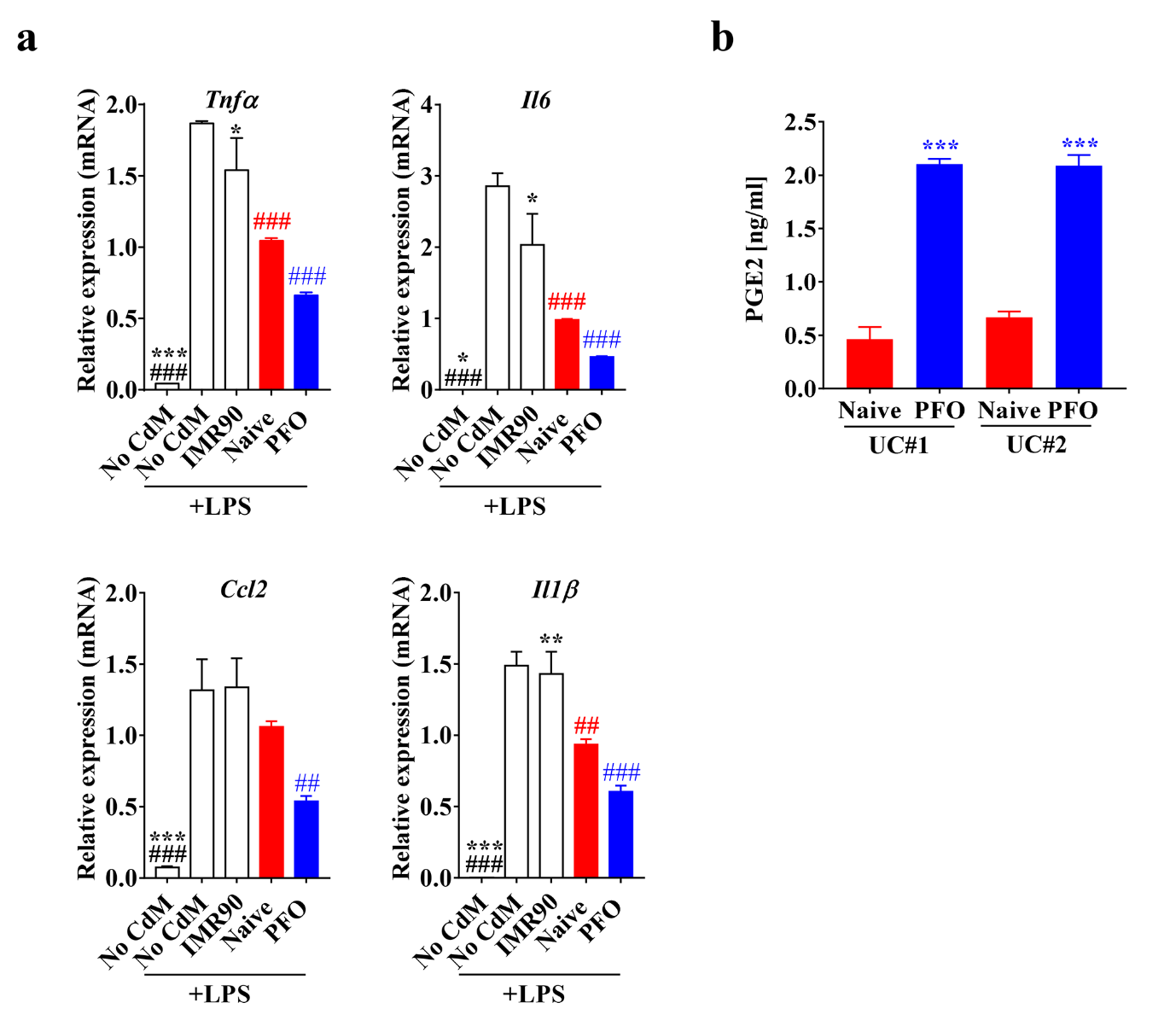
**

**Supplementary Fig. S7. Effects of the PFO procedure on the anti-inflammatory properties of human UC-MSCs**

**(a)** Quantitative levels of expression of transcripts of pro-inflammatory cytokines by LPS-stimulated MH-S in the absence (No CdM) or presence of CdM harvested from the indicated cells. Quantitation of the secretion of these pro-inflammatory cytokine proteins are shown in **Figure 2e**. **(b)** Levels of prostaglandin E2 (PGE2) in the CdM of naïve and PFO UC-MSCs, the latter generated by AA2G supplemention for 7 days. All quantitative data are reported as mean ± SEM (*n*=4 for each donor MSCs) ratios relative to naïve MSCs and analyzed by one-way ANOVA with Bonferroni *post-hoc* tests. **P* < 0.05, ***P* < 0.01, ****P* < 0.001 compared with naïve cells. ##*P* < 0.01, ###*P* < 0.001 compared with LPS-stimulated MH-S in the absence of CdM (No CdM).

**
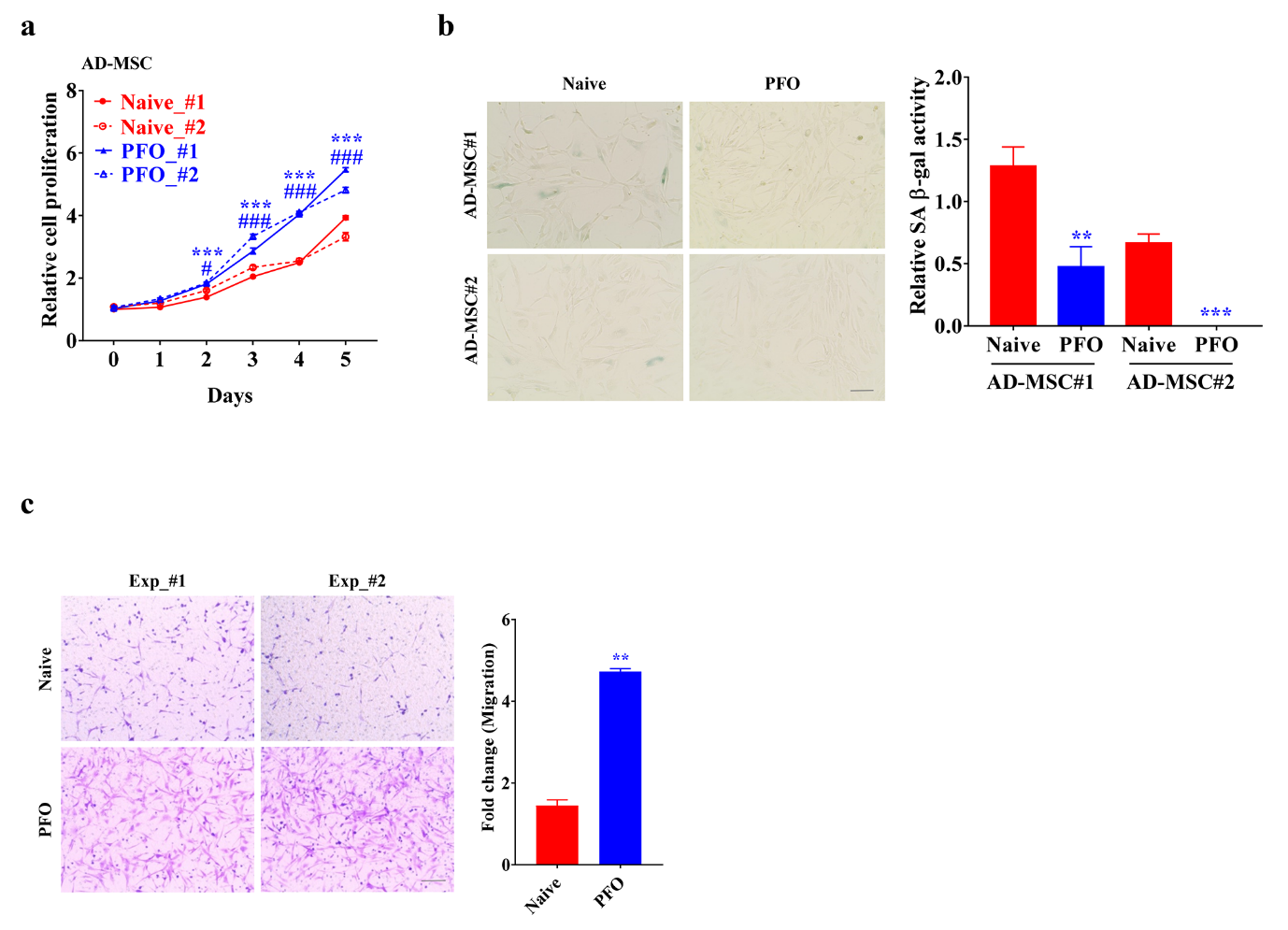
**

**Supplementary Fig. S8. Beneficial effects of the PFO procedure on human AD-MSCs**

**(a−c)** Cell proliferation, as determined by MTT assays, on the indicated days **(a,** *n*=5**)**, SA β-gal staining **(b,** *n*=7**)**, and chemotactic **response** to PDGF (**c,** *n*=6) of naïve and PFO human AD-MSCs from two independent donors generated by AA2G treatment for 7 days. Quantitative results for each donor MSC are presented as the mean ± SEM relative to naïve MSCs. **(a)** ****P* < 0.001 compared with donor #1; and #*P* < 0.05, ###*P* < 0.001 compared with donor #2 by two-way ANOVA with Bonferroni *post-hoc* tests. **(b and c)** ***P* < 0.01, ****P* < 0.001 compared with naïve MSCs by one-way ANOVA **(b)** or non-parametric Mann–Whitney U tests **(c)**. Representative images for each assay are presented in the left panel and shown at × 200 magnification (scale bar=100 μm).


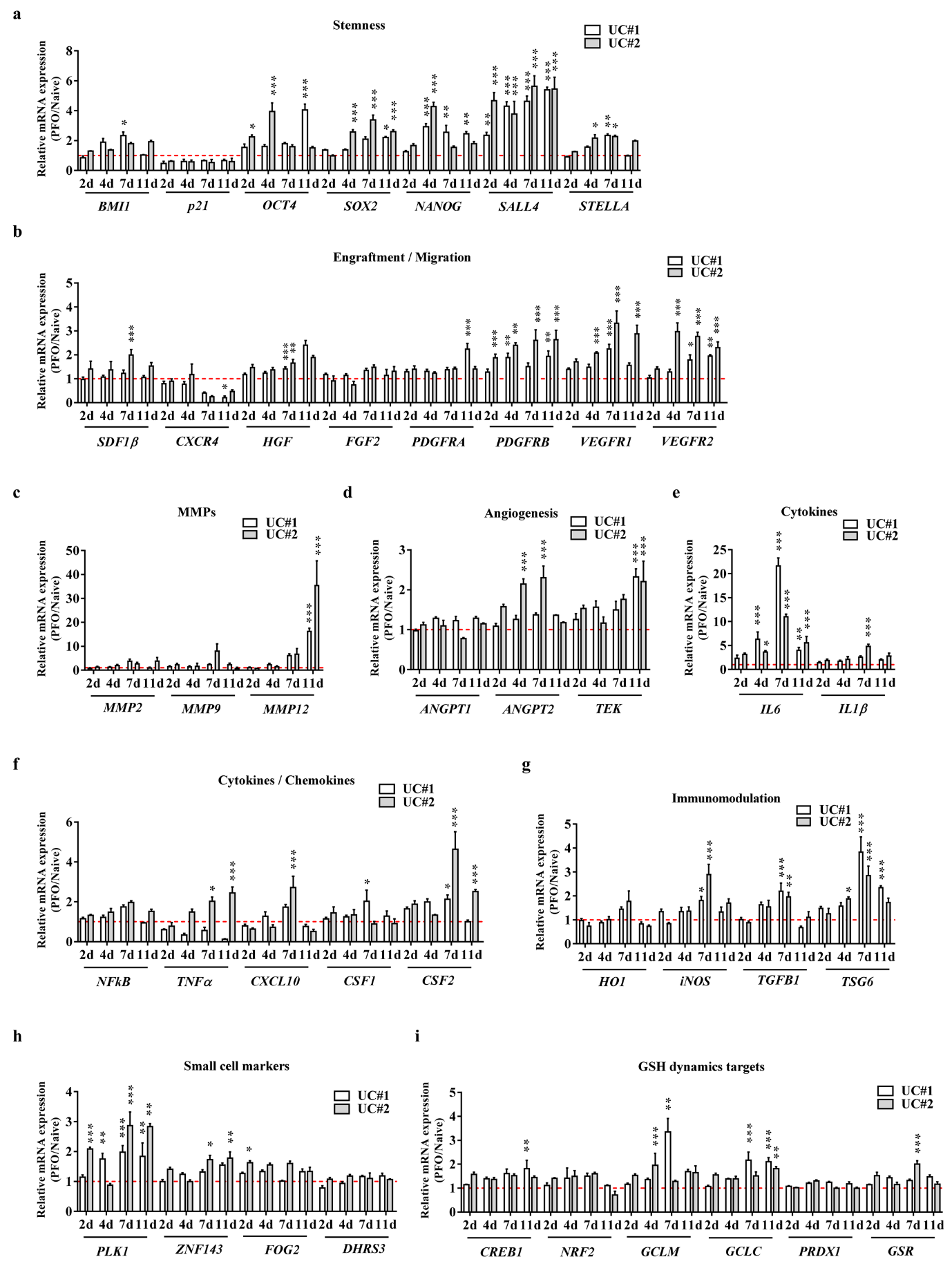


**Supplementary Fig. S9. Gene expression profiles of PFO UC-MSCs**

**(a−i)** Quantitative analysis of the expression of the indicated genes in naïve and PFO UC-MSCs, generated by AA2G treatment for the indicated number of days, from two independent donors (UC#1 and UC#2), as shown by RQ-PCR assays. Levels of expression are shown as fold changes relative to naïve MSCs. Source data are available in **Supplementary Dataset S1**. Validation of changes in expression by quantitative analysis of genes related to stemness **(a)**, engraftment and migration **(b)**, matrix metalloproteinases (MMPs) **(c)**, angiogenesis **(d)**, cytokines and chemokines **(e and f)**, immune-modulatory mediators of MSCs **(g)**, and markers for small-sized MSCs **(h)** enriched by filtration through a pluriStrainer with a pore size of 10 μm, followed by hypoxic conditions,^6^ as well as CREB1−NRF2 dependent target genes **(i)** for modulating GSH dynamics.^4^ Levels of expression are reported as the mean ± SEM (*n*=4 for each donor MSC) relative to naïve MSCs for each duration of AA2G treatment. **P* < 0.05, ***P* < 0.01, ****P* < 0.001 compared with naïve cells by two-way ANOVA with Bonferroni *post-hoc* tests.


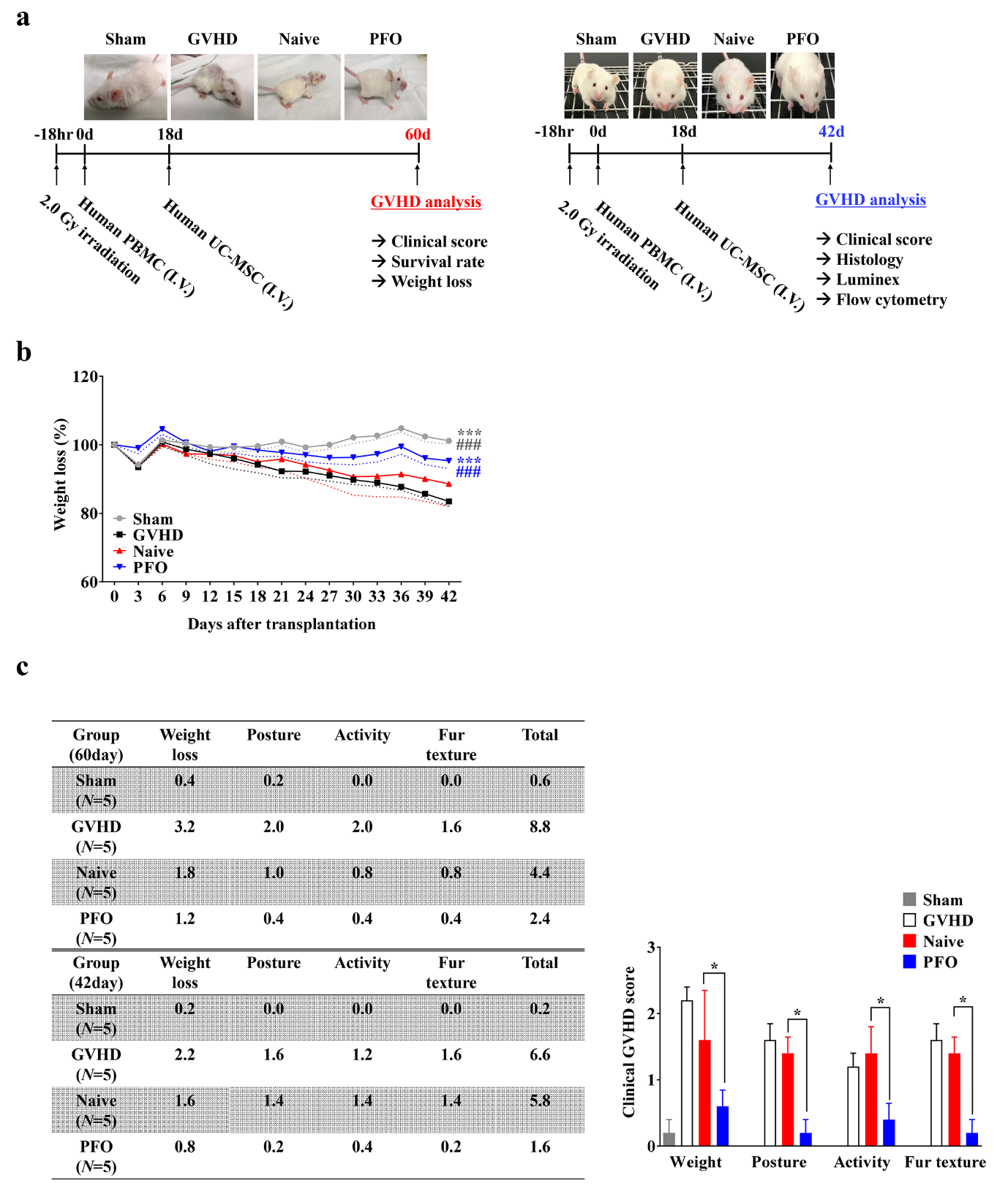


**Supplementary Fig. S10. Enhanced *in vivo* therapeutic efficacy and engraftment of PFO UC-MSCs for treating GVHD**

**(a)** Schematic overview of the experimental protocols for the induction of GVHD in the two sets of mice. Non-obese diabetic (NOD).Cg-*Prkdc*^scid^ *Il2rg^tm1Wjl^*/SzJ (NSG) mice were injected via the tail vein with 1.0×10^6^ human peripheral blood mononuclear cells (PBMCs), followed by 1×10^5^ naïve human UC-MSCs or cells subjected to the PFO procedure 18 days after GVHD induction (arrow). In the first set (five animals per group), mouse survival and weight loss were assessed for 60 days after PBMC infusion. In the second set (five mice per group), the animals were sacrificed 42 days after administration of human PBMCs. GVHD target organs were assessed histologically, donor T cell populations were analyzed immunologically, and multiplex human cytokine assays were performed. **(b)** Body weight loss in the second set of GVHD mice (*n*=5 per group) of animals recorded every second day for 42 days after injection of human PBMCs injection before sacrifice. Quantitative results (*n*=5) are presented as mean (solid line) ± SEM (dotted line). **(c and d)** GVHD clinical scores in the first and second sets of animals. GVHD clinical score was based on weight loss (1, 1–10%; 2, 11–20%; 3, 21–30%; and 4, 31–40%), posture, activity, and fur texture (0, normal; 1, mildly abnormal; and 2, severely abnormal). All groups were evaluated every second day, with the scores shown in the table **(c)** and bar graph **(d)** being the averages on day 42 (*n*=5). **P* < 0.05 compared with the naïve group by two-way ANOVA with Bonferroni *post-hoc* tests.


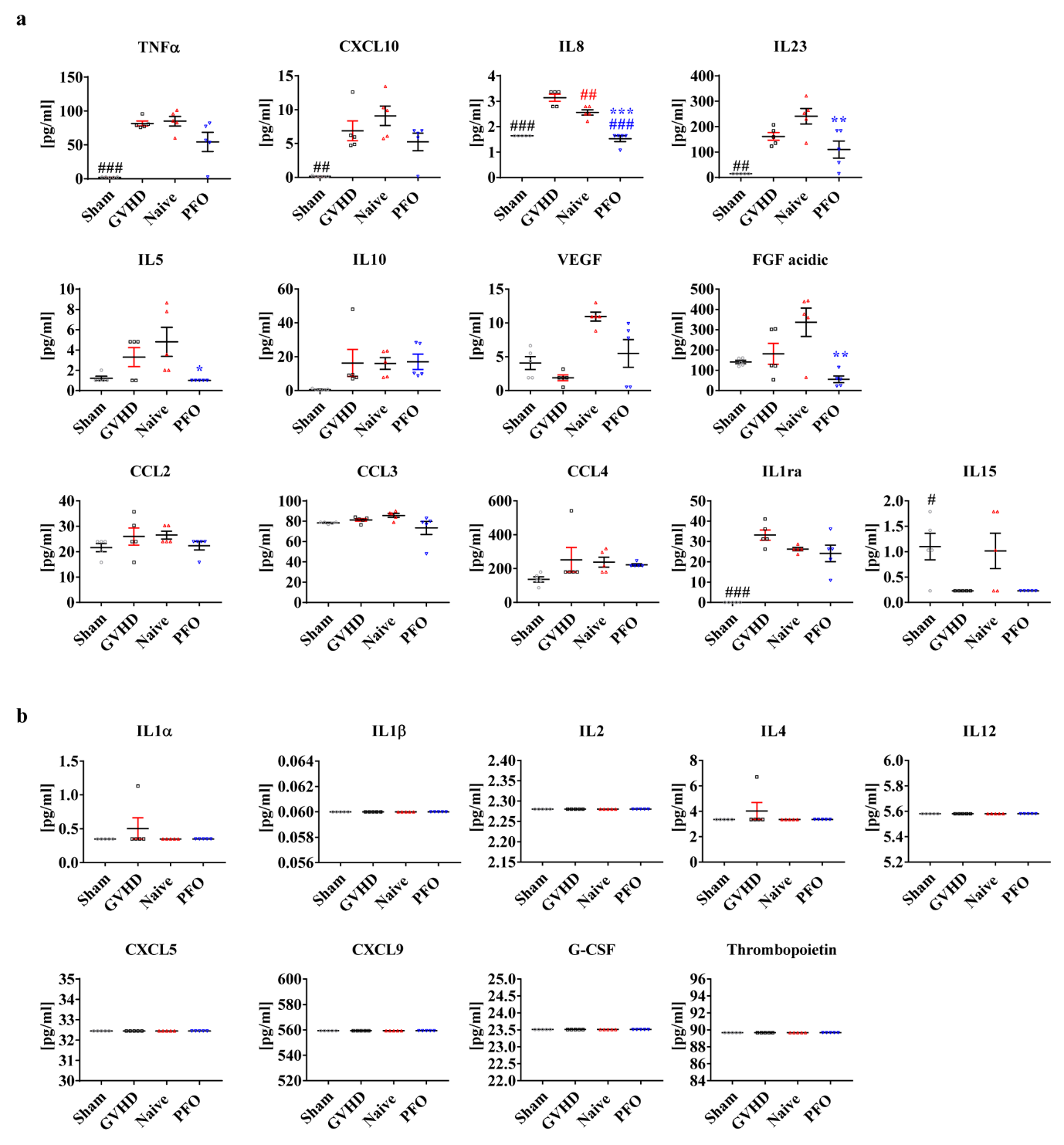


**Supplementary Fig. S11. Cytokine profiles in the sera of GVHD mice**

**(a)** Multiplex analysis of the indicated human cytokines and chemokines in sera from GVHD mice 6 weeks after infusion of human PBMCs. Data are presented as mean ± SEM (*n*=5) and compared by one-way ANOVA. **P* < 0.05, ***P* < 0.01, ****P* < 0.001 compared with the naïve group; ##*P* < 0.01, ###*P* < 0.001 compared with the GVHD group. **(b)** Cytokines and chemokines expressed at low levels in all tested groups.

**
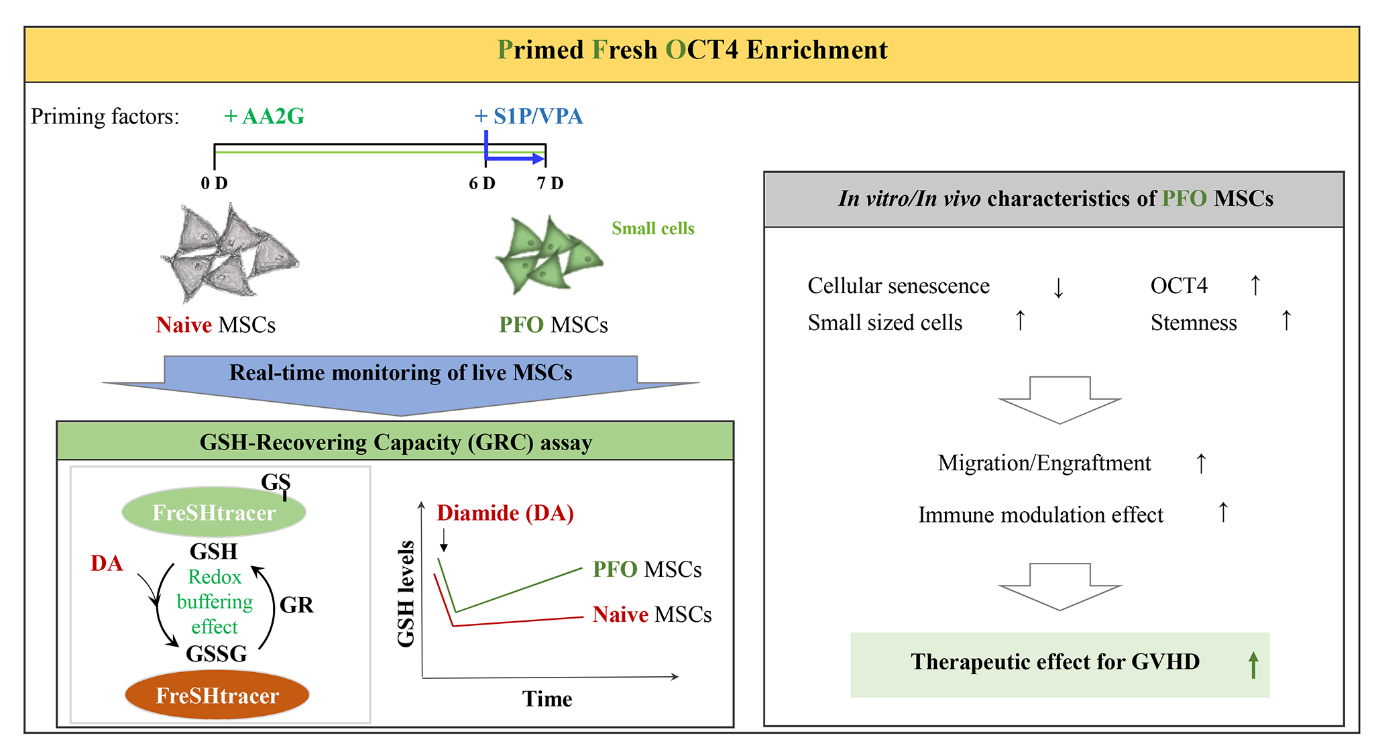
**

**Supplementary Fig. S12. Graphic abstract**

This study describes an optimal environment for preserving primitive MSCs, characterized morphologically by small size and with high glutathione (GSH) dynamics. MSCs were cultured in medium containing ascorbic acid 2-glucoside (AA2G) and low concentrations of sphingosine 1-phosphate (S1P) and valproic acid (VPA). Primitive MSCs enriched by the **P**rimed **F**resh **O**CT-4 (PFO) enrichment procedure showed enhanced core functions and greater therapeutic efficacy in the treatment of graft-versus-host disease (GVHD). To evaluate GSH recovery capacity (GRC), an indicator of cellular antioxidant capacity, GSH changes in every living single cell were traced in real-time using an Operetta high-content imaging analysis system and FreSHtracer (**F**luorescent **re**al-time **thiol** tracer), a reversible GSH fluorescent probe.^4,7^


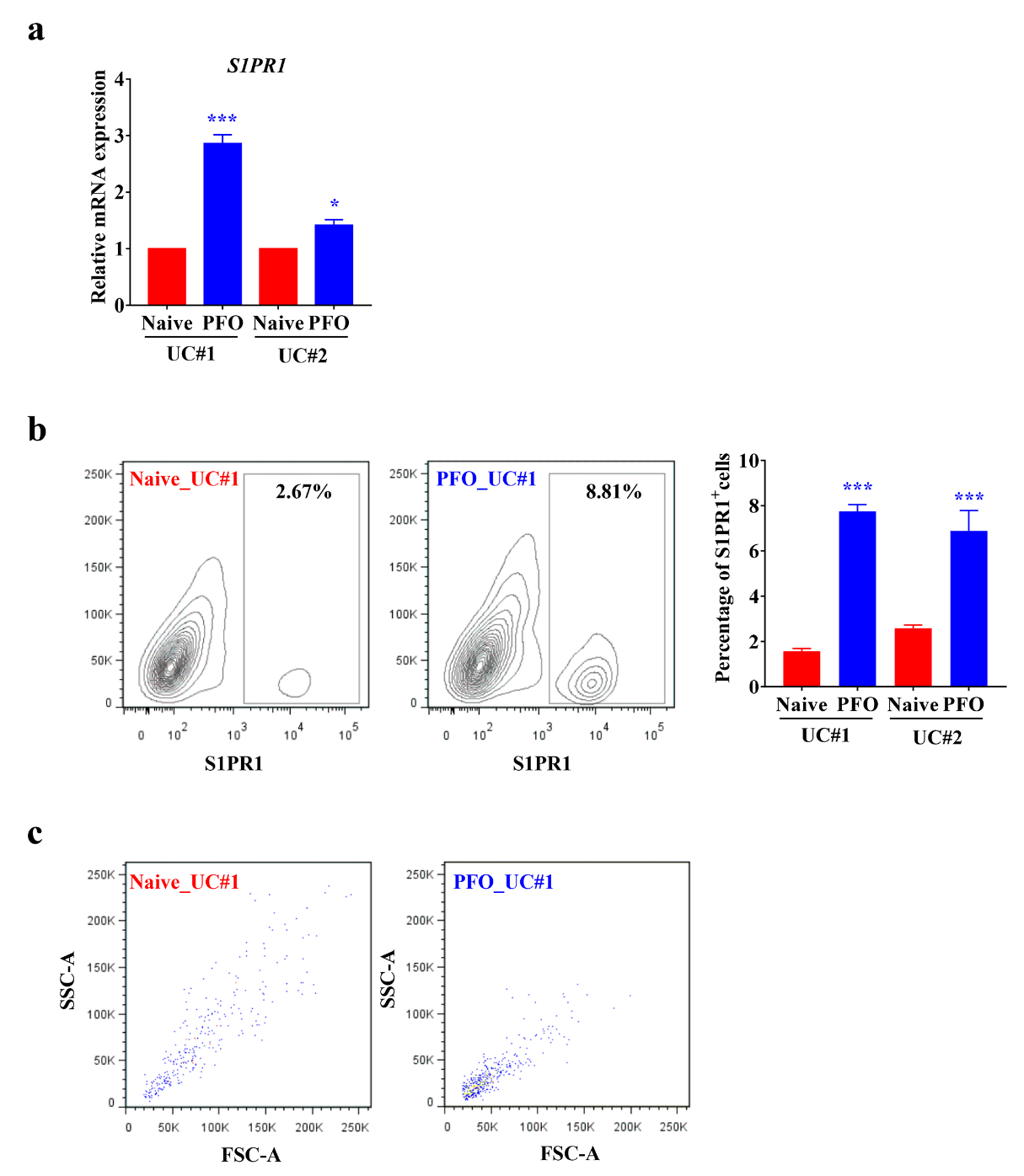


**Supplementary Fig. S13. Up-regulation of S1PR1 by the PFO procedure**

**(a)** Real-time quantitative PCR (RQ-PCR) assays for the expression of sphingosine-1-phosphate receptor-1 (S1P receptor-1; *S1PR1*) in normal (naïve) cultures of human UC-MSCs and in cells subjected to the PFO procedure by culture in the presence of AA2G for 7 days. **(b)** Flow cytometric analysis of S1PR1 protein expression in naïve and PFO UC-MSCs from two independent donors (UC#1 and UC#2). The percentage of S1PR1^+^ cells is shown on the right side of the cytogram and presented as mean ± SEM (*n*=4 for each donor). ****P* < 0.001 compared with the naïve group by one-way ANOVA with Bonferroni *post-hoc* tests. **(c)** Representative flow cytometric analysis of the sizes of S1PR1^+^ cells in populations of naïve and PFO UC-MSCs.

**SUPPLEMENTARY NOTES**

Several immunomodulatory cells including MSCs have been investigated as potential targets for treating GVHD, a major complication of allogeneic hematopoietic stem cell transplantation. However, loss of primitive states of MSCs after *ex vivo* expansion has yielded seemingly conflicting preclinical and clinical results on the effects of MSC-based therapies.

Primitive MSCs have been reported to be enriched by cell size filtration,^6^ flow cytometric cell sorting based on high anti-oxidant capacity,^7^ and genetic modulation.^4^ Although these strategies showed *in vivo* proof of concept and provided related mechanistic insights, they still exhibited critical drawbacks for clinical applications, particularly regarding the low yields of primitive MSCs following enrichment. For example, the Small cells primed with Hypoxia and Calcium ions (SHC) procedure showed a >60% loss of cells by initial filtration through a pluriStrainer with a pore size of 10 μm.^6^ Flow cytometric sorting of MSCs with higher GSH level using FreSHtracer, a reversible chemical probe for GSH,^4,7^ found that only around 10% of the initial cells could be obtained as primitive MSCs. Although GSH dynamics can be enhanced by ectopic expression of specific CREB1−NRF2 target genes,^4^ lentiviral gene delivery is not sufficiently safe for practical application in clinical settings.

The present study showed that a simple combination of three small molecules, AA2G, S1P, and VPA, provided an optimal environment for *in vitro* capture and stable expansion of primitive MSCs, with little cell loss and few of the safety concerns associated with genetic manipulation. More importantly, this PFO procedure recapitulated the beneficial effects of the aforementioned strategies for enriching primitive MSCs,^4,6,7^ characterized by small size and high anti-oxidant capacity **(Supplementary Fig. 12)**. These activities critically influenced the developmental potency and therapeutic efficacy of MSCs. Indeed, human PFO/UC-MSCs exhibited enhanced stemness and immunomodulatory effects for treating allogeneic conflicts, as demonstrated using cell culture-based assays **(Figure 2)** and a humanized GVHD mouse model **(Figure 3)**. Additional studies should investigate the detailed molecular signature and homogeneity of PFO MSCs by methods that include multi-omics and single cell analysis, respectively.

Small-sized stem cells are regarded as the most primitive population of stem cells, which could play a role in orchestrating homeostasis and repair processes of adult tissues. For example, multilineage-differentiating stress-enduring (Muse) cells^16-18^ and very small embryonic-like stem cells (VSELs)^19-22^ can differentiate into cells of all three germ layers *in vitro* and in animal models of tissue regeneration. These cells consistently exhibit the molecular characteristics of pluripotent stem cells (PSCs). In particular, VSELs derived from adult mouse bone-marrow exhibit unique epigenetic features, including DNA demethylation in the promoter of *Oct4* and germline lineage genes as well as the differentially methylated regions (DMRs) of a subset of imprinted genes related to insulin/insulin-like growth factor signaling (*Igf2*, *H19*, *Igf2R*, and *Rasgrf1*).^23-25^

Molecular analysis showed that the primitive small sized UC-MSCs enriched by the PFO procedure shared several molecular features with VSELs. Compared with naïve MSCs, PFO UC-MSCs exhibited increased expression of PSC markers, including *OCT4* and *SOX2* **(Figure 1g)**. In addition, the *OCT4* promoter in PFO UC-MSCs showed an open/active chromatin structure characterized by DNA demethylation **(Figure 1i)** and enrichment with active histone codes, including the acetylation and the trimethylated lysine-4 of histone H3 **(Supplementary Fig. S6a)**. Moreover, the up-regulated OCT4 protein was found to localize to the nuclei of small sized UC-MSCs enriched by the PFO procedure, indicating the expression of OCT4-A protein,^26^ which is specific to PSCs (**Figure 1h**). Furthermore, PFO UC-MSCs exhibited DNA demethylation in the DMR of the *H19* locus **(Supplementary Fig. S6b)**, a molecular signature of VSELs.^23-25^ These results indicate that small sized cells enriched by the PFO procedure are strongly related to VSELs. Additional studies comparing the molecular features of PFO MSCs and small primitive stem cells, including VSELs, might advance understanding about the molecular nature of small primitive stem cells deposited in adult tissues, and may lead to the elucidation of their developmental hierarchy. In addition, the PFO procedure may be beneficial in enriching and maintaining most primitive stem cells deposited in adult tissues, including VSELs, which are quiescent and difficult to expand *ex vivo*. Because the PFO procedure up-regulated sphingosine-1-phosphate receptor-1 (*S1PR1*) mRNA and protein (**Supplementary Fig. 13**), studies are needed to assess the expression and function of S1PR1 on small-sized stem cells (e.g., PFO MSC, VSELs, and Muse cells).

A series of *in vitro* cellular assays showed that the PFO procedure enhanced core functions of MSCs, including stemness, migration, anti-inflammatory, and immunomodulatory activities (**Figure 2**). Because the PFO procedure is a reliable method for optimizing the cost-effective and safe (without genetic manipulation) *ex vivo* expansion of functionally qualified MSCs, this procedure may improve the efficacy of MSC-based therapies in patients with incurable GVHD. Indeed, administration of human UC-MSCs subjected to the PFO procedure significantly ameliorated symptoms of GVHD in the humanized GVHD mouse model, resulting in significantly improved survival, less weight loss, and reduced histopathologic injuries in GVHD target organs compared with naïve MSC-infused mice (**Figure 3**). Therefore, the present study provides further evidence for the molecular and functional significance of small size and GSH dynamics of MSCs in determining the outcomes of treatment for GVHD.

The animal model of GVHD tested in this study utilized human PBMCs isolated from healthy donors as effector cells. Moreover, because immunocompromised NSG mice exhibit reduced activities of T-cells, B-cells, and NK cells, they were used as recipients to ensure high levels of engraftment of human PBMCs.^27^ Human MSCs allogenic to the PBMC donor were intravenously injected 18 days after human PBMC infusion into NSG mice preconditioned by exposure to 2.0 Gy irradiation (**Supplementary Fig. S10a**). Thus, this humanized GVHD model recapitulates the clinical setting to evaluate the mechanistic and therapeutic outcomes of batches of MSCs against donor lymphocytes. However, the immune reaction in a xenogeneic setting may be different than the allogenic response in human patients with GVHD. Indeed, the absence of human stromal factors from this NSG mouse model may limit the expansion of human regulatory T cells, which is a potential mode of action of MSC therapy.^27^ Although *in vitro* MLR assays showed that the immunomodulatory activity of PFO UC-MSCs in response to allogeneic conflicts was enhanced (**Figure 2h**), the beneficial effects of the PFO procedure should be further validated in an allogenic mouse GVHD model. More importantly, careful investigation of the *in vivo* mode of action and clinical relevance of MSCs expanded by the PFO procedure is required to successfully translate these promising preclinical results from a xenograft model into clinical practice.

The limitations of the GVHD animal model used in this study should be discussed in depth. This study utilized two independent sets of animals (**Supplementary Fig. S10a**). In the first set, mouse survival and weight loss were examined for 60 days after administration of human PBMCs. In the second set, the animals were sacrificed 42 days after infusion of human PBMCs. GVHD target organs were assessed histologically, donor human T cell populations were determined in spleen, and multiplex human cytokine profiles in serum were examined to investigate the mechanisms underlying the effects of MSCs. The small number of animals in each set (*n*=5 per group) limited the significance of these *in vivo* results. For example, the administration of naïve or PFO UC-MSCs significantly improved the survival rates of GVHD mice, with survival rates being higher in mice treated with PFO UC-MSCs than with naïve cells. Because of the small number of animals, however, the latter difference did not reach the threshold for statistical significance (**Figure 3a**). Despite this limitation, PFO UC-MSCs, when compared with naïve cells, significantly ameliorated the symptoms of GVHD, including weight loss, clinical scores (based on body weight loss, survival, hunched back posture, and fur texture; **Supplementary Fig. 10**), histological injuries to representative GVHD target organs (small intestine, lungs, liver, and kidneys), and immunological responses based on the immunological analyses of donor T cell populations in spleen, and multiplex human cytokine analysis in serum (**Figure 3b−3c**). Collectively, these results demonstrate that the PFO procedure enhances the therapeutic potency of UC-MSCs for treating GVHD.

The present study also had several other limitations. For example, injury to the skin, a major target organ of GVHD, was not investigated, nor was the status of donor derived human lymphocytes in peripheral blood or lymph nodes of GVHD mice. In addition, although the treatment efficacy of PFO UC-MSCs was evaluated *in vivo*, their ability to prevent GVHD symptoms *in vivo* efficacy was not, nor were the anti-tumor and anti-pathogen responses to MSC therapy. Although MSC therapy contributes to the control of allogenic conflicts by modulating the apoptosis and proliferation of donor T cells *in vivo,*^27^ these important modes of action of MSC therapy were not examined in the GVHD animal model. Additional studies are therefore necessary to provide greater insight into the mechanisms by which the PFO procedure enhances the therapeutic activities of MSCs, enabling the clinical use of PFO UC-MSCs.

Although advances have improved patient survival after allogeneic hematopoietic stem cell transplantation, GVHD remains a leading cause of late morbidity and mortality. Unfortunately, effective second-line treatments and standards of care are lacking for patients refractory to first-line steroid therapy.^28^ MSC-based therapies are being developed and yielding exciting results.^29,30^ However, the high cost and lack of a standardized preparation procedure limit the use of MSCs as first-line treatment for established GVHD.^31,32^ The PFO procedure presented in this study advances the previous strategies for *in vitro* capture and *ex vivo* expansion of primitive MSCs. This procedure may stably provide functionally qualified MSCs in a cost-effective and safe (without genetic manipulation) manner, improving the results of future clinical trials of MSCs for treating GVHD and other intractable immune-related disorders, including systemic lupus erythematosus and asthma.

**TABLE S1 (KEY RESOURCE TABLE)**

| **REAGENT or RESOURCE** | **SOURCE** | **IDENTIFIER** |
| --- | --- | --- |
| **Antibodies** | | |
| NRF2 | Abcam | Ab62352 |
| p53 | Santa Cruz Biotechnology | SC6243 |
| BMI1 | Active motif | #39994 |
| human CD3 | BD Biosciences | #300412 |
| human CD4 | BD Biosciences | #300512 |
| human CD14 | BD Biosciences | #555397 |
| human CD29 | BD Biosciences | #555443 |
| human CD34 | BD Biosciences | #555822 |
| human CD45 | BD Biosciences | #555483 |
| mouse CD45 | BD Biosciences | #553079 |
| human CD49f | BD Biosciences | #555735 |
| human CD73 | BD Biosciences | #550257 |
| human CD90 | BD Biosciences | #555596 |
| human CD105 | BD Biosciences | #560819 |
| Human CD363 (S1PR1) | Invitrogen | #50-3639-42 |
| Human β-2-microglobulin | Santa Cruz Biotechnology | SC80668 |
| H3K4me3 | Abcam | Ab8580 |
| H3K27me3 | Millipore | #07-449 |
| H3Ac | MERCK | #06-599 |
| OCT4 | Millipore | MAB4419 |
| SOX2 | Abcam | Ab92494 |
| Anti-mouse IgG, Alexa Fluor 488 | Thermo Fisher Scientific | A-11001 |
| Anti-rabbit IgG, Alexa Fluor 488 | Thermo Fisher Scientific | A-11008 |
| **Biological Samples** | | |
| Human peripheral mononuclear cells | Stem Cell Technologies | #70025 |
| **Chemicals, Peptides, and Recombinant Proteins** | | |
| FBS | Hyclone | SH30084.03 |
| DMEM-high-glucose medium | Hyclone | SH30243.01 |
| DMEM-low-glucose medium | Hyclone | SH30021.01 |
| DMEM/F12 medium | Hyclone | SH30261.01 |
| L-glutamine | Corning | 26-065-CI |
| HEPES | Corning | 25-060-CI |
| MEM nonessential amino acid solution | Corning | 25-025-CIR |
| Penicillin/streptomycin solution | Corning | 30-002-CI |
| Human epidermal growth factor | ProSpec | CYT-218 |
| Basic fibroblast growth factor | ProSpec | CYT-002 |
| Ascorbic acid | Sigma-Aldrich | A4544 |
| PDGF-AA | R&D Systems | #221-AA |
| DNase I | Sigma-Aldrich | M8823 |
| 4% Paraformaldehyde | Sigma-Aldrich | P6148 |
| RIPA lysis buffer | Santa Cruz Biotechnology | sc-24948 |
| Protease inhibitor cocktails | Roche | #05892791001 |
| Phosphatase inhibitor cocktails | Roche | #04906837001 |
| Sodium butylate | Millipore | #19-137 |
| Diamide | Sigma-Aldrich | D3648 |
| RNeasy purification kit | QIAGEN | #74104 |
| Valproic acid | Sigma-Aldrich | P4543 |
| Ascorbic acid 2-glucoside | MERCK | #SMB00390 |
| Sphingosine 1-phosphate | MERCK | #73914 |
| Crystal violet | Sigma-Aldrich | C0775 |
| Phytohemagglutinin | Sigma-Aldrich | L8754 |
| CFSE cell division tracker kit | BioLegend | #423801 |
| Lipopolysaccharide | Sigma-Aldrich | L3012 |
| **Critical Commercial Assays** | | |
| Senescence β-galactosidase staining kit | Cell Signaling Technology | #9860 |
| Magnetic Luminex Screening Assay human Premixed Multi-Analyte Kit | R&D System | LXSAHM-28 |
| Human Prostaglandin E_2_ ELISA Kit | Invitrogen | KHL1701 |
| Cell Proliferation ELISA, BrdU | Roche | #11647229001 |
| Mouse Tnfα ELISA | Ab Frontier | LF-EK0275 |
| Mouse Il6 ELISA kit | Invitrogen | BMS603-2 |
| Mouse Mcp1 ELISA kit | Invitrogen | BSM6005 |
| Magna ChIP G kit | Millipore | MAGNA0002 |
| **Experimental Models: Cell Lines** | | |
| IMR90 | ATCC | CCL-186 |
| 293FT | ThermoFisher Scientific | R70007 |
| Human UC-MSC | This study | N/A |
| Human AD-MSC | This study | N/A |
| **Experimental Models: Organisms/Strains** | | |
| NOD.Cg-*Prkdc^scid^* *Il2rg^tm1Wjl^*/SzJ (NSG) | The Jackson Laboratory | JAX#005557 |
| **Oligonucleotides (qPCR)** | | |
| human *CREB1* Forward  GCCACTCAGCCGGGTACTAC | Lim et al. 2020 | N/A |
| human *CREB1* Reverse  AACTTGGTTGCTGGGCACTAA | Lim et al. 2020 | N/A |
| human *NRF2* Forward  TGAGGATTCCTTCAGCAGCAT | Lim et al. 2020 | N/A |
| human *NRF2* Reverse  GACTGTGGCATCTGAATTTAATGAGT | Lim et al. 2020 | N/A |
| human *GCLC* Forward  GGAGGAAACCAAGCGCCAT | Lim et al. 2020 | N/A |
| human *GCLC* Reverse  CTTGACGGCGTGGTAGATGT | Lim et al. 2020 | N/A |
| human *GCLM* Forward  TGTCTTGGAATGCACTGTATCTC | Lim et al. 2020 | N/A |
| human *GCLM* Reverse  CCCAGTAAGGCTGTAAATGCTC | Lim et al. 2020 | N/A |
| human *GSR* Forward  TTCCAGAATACCAACGTCAAAGG | Lim et al. 2020 | N/A |
| human *GSR* Reverse  GTTTTCGGCCAGCAGCTATTG | Lim et al. 2020 | N/A |
| human *PRDX1* Forward  CATTCCTTTGGTATCAGACCCG | Lim et al. 2020 | N/A |
| human *PRDX1* Reverse  CCCTGAACGAGATGCCTTCAT | Lim et al. 2020 | N/A |
| human *DHRS3* Forward  ACTGAGTGCCATTACTTCATCTG | Kim et al. 2018 | N/A |
| human *DHRS3* Reverse  CATCACTGTCCATTAGGCTCTTC | Kim et al. 2018 | N/A |
| human *PLK1* Forward  AAAGAGATCCCGGAGTCCTA | Kim et al. 2018 | N/A |
| human *PLK1* Reverse  GGCTGCGGTGAATGGATATTTC | Kim et al. 2018 | N/A |
| human *ZFP143* Forward  GTACAGGGGACAGTTTGCGTC | Kim et al. 2018 | N/A |
| human *ZFP143* Reverse  TGGAGGTGTGGTGAATAAATGC | Kim et al. 2018 | N/A |
| human *FOG2* Forward  ATGTCCCGGCGAAAGCAAA | Kim et al. 2018 | N/A |
| human *FOG2* Reverse  AGCTCAGATTTTCAGGCCCAA | Kim et al. 2018 | N/A |
| human *OCT4* Forward  GAGCCCTGCACCGTCACC | This study | N/A |
| human *OCT4* Reverse  TTGATGTCCTGGGACTCCTCC | This study | N/A |
| human *SOX2* Forward  TACAGCATGTCCTACTCGCAGC | This study | N/A |
| human *SOX2* Reverse  GAGGAAGAGGTAACCACAGGGG | This study | N/A |
| human *NANOG* Forward  GCAGAAGGCCTCAGCACCTA | This study | N/A |
| human *NANOG* Reverse  AGGTTCCCAGTCGGGTTCA | This study | N/A |
| human *SALL4* Forward  CCAGGGAATGACGAGGTGG | This study | N/A |
| human *SALL4* Reverse  GAACTCCGCACAGCATTTCTC | This study | N/A |
| human *BMI1* Forward  CGTGTATTGTTCGTTACCTGGA | This study | N/A |
| human *BMI1* Reverse  TTCAGTAGTGGTCTGGTCTTGT | This study | N/A |
| human *p21* Forward  TGTCCGTCAGAACCCATGC | This study | N/A |
| human *p21* Reverse  AAAGTCGAAGTTCCATCGCTC | This study | N/A |
| human *SDF1β* Forward  TGCGTCCACGAGCTGTTTAC | This study | N/A |
| human *SDF1β* Reverse  CCCAAGGGAGTGTCAGGTAGAG | This study | N/A |
| human *CXCR4* Forward  ACTACACCGAGGAAATGGGCT | This study | N/A |
| human *CXCR4* Reverse  CCCACAATGCCAGTTAAGAAGA | This study | N/A |
| human *cMET* Forward  AGCGTCAACAGAGGGACCT | This study | N/A |
| human *cMET* Reverse  GCAGTGAACCTCCGACTGTATG | This study | N/A |
| human *HGF* Forward  CTCACACCCGCTGGGAGTAC | This study | N/A |
| human *HGF* Reverse  TCCTTGACCTTGGATGCATTC | This study | N/A |
| human *PDGFRA* Forward  TTGAAGGCAGGCACATTTACA | This study | N/A |
| human *PDGFRA* Reverse  GCGACAAGGTATAATGGCAGAAT | This study | N/A |
| human *PDGFRB* Forward  TGATGCCGAGGAACTATTCATCT | This study | N/A |
| human *PDGFRB* Reverse  TTTCTTCTCGTGCAGTGTCAC | This study | N/A |
| human *VEGFR1* Forward  CTCTCTCCCTGATCGGTGACA | This study | N/A |
| human *VEGFR1* Reverse  GGAGGGCAGAGCTGAGTGTTAG | This study | N/A |
| human *VEGFR2* Forward  GGTTGCATTACTGTACCCATCATTT | This study | N/A |
| human *VEGFR2* Reverse  TGAGATGGAATCTGACCATGTTG | This study | N/A |
| human *MMP2* Forward  TGATGGTTCCCCTGTTCACTCTA | This study | N/A |
| human *MMP2* Reverse  GCCACGGCTTGGTTTTCCT | This study | N/A |
| human *MMP9* Forward  CCCGGAGTGAGTTGAACCA | This study | N/A |
| human *MMP9* Reverse  CAGGACGGGAGCCCTAGTC | This study | N/A |
| human *MMP12* Forward  GATCCAAAGGCCGTAATGTTCC | This study | N/A |
| human *MMP12* Reverse  TGAATGCCACGTATGTCATCAG | This study | N/A |
| human *ANGPT1* Forward  TGCTCACGTGGCTCGACTATA | This study | N/A |
| human *ANGPT1* Reverse  AGCACAGCAAGCTCAGCAGTTT | This study | N/A |
| human *ANGPT2* Forward  GGTTTGATGCATGTGGTCCTT | This study | N/A |
| human *ANGPT2* Reverse  AATGCCGTTGAACTTATTTGTGTTC | This study | N/A |
| human *IDO1* Forward  TCCGTGAGTTTGTCCTTTCAAA | This study | N/A |
| human *IDO1* Reverse  CAGGGAGACCAGAGCTTTCACA | This study | N/A |
| human *IDO2* Forward  GATTGATGCTCACCAGCTTCAAG | This study | N/A |
| human *IDO2* Reverse  GCTCCCGGTGACCCTTCAG | This study | N/A |
| human *TIE1* Forward  CCTGTGCCGAGCTCTATGAAAA | This study | N/A |
| human *TIE1* Reverse  GCTCGTACACTTCATCGTCACAGT | This study | N/A |
| human *TEK* Forward  GGAGACGGACCCAGCATTT | This study | N/A |
| human *TEK* Reverse  CGGCAGCGAAGTGAAGGA | This study | N/A |
| human *LIF* Forward  GAAAGCTTTGGTAGGTTCTTCGTT | This study | N/A |
| human *LIF* Reverse  TGCAGGTCCAGCCATCAGA | This study | N/A |
| human *NFkB* Forward  AACAGAGAGGATTTCGTTTCCG | This study | N/A |
| human *NFkB* Reverse  TTTGACCTGAGGGTAAGACTTCT | This study | N/A |
| human *TNFα* Forward  GCCAGGCAGGTTCTCTTCCT | This study | N/A |
| human *TNFα* Reverse  TCAGTGCTCATGGTGTCCTTTC | This study | N/A |
| human *IL6* Forward  TCATTCTGCGCAGCTTTAAGG | This study | N/A |
| human *IL6* Reverse  CAATCTGAGGTGCCCATGCT | This study | N/A |
| human *IL1β* Forward  GACAGAAACCACGGCCACAT | This study | N/A |
| human *IL1β* Reverse  TAGGGAAGCGGTTGCTCATC | This study | N/A |
| human *IL12a* Forward  TTCAGAATTCGGGCAGTGACT | This study | N/A |
| human *IL12a* Reverse  CCCCCTCCCTAGTTCTTAATCC | This study | N/A |
| human *IL12b* Forward  GCTATGGTGAGCCGTGATTGT | This study | N/A |
| human *IL12b* Reverse  GCCATGGAAGCTAAAGCTGAA | This study | N/A |
| human *CXCL10* Forward  GTGGCATTCAAGGAGTACCTC | This study | N/A |
| human *CXCL10* Reverse  TGATGGCCTTCGATTCTGGATT | This study | N/A |
| human *CSF1* Forward  TGCTGGAGAAGGTCAAGAATGTC | This study | N/A |
| human *CSF1* Reverse  GTTGTTGCAGTTCTTGCTGAAAA | This study | N/A |
| human *CSF2* Forward  AGCCCTGGGAGCATGTGA | This study | N/A |
| human *CSF2* Reverse  ATTCATCTCAGCAGCAGTGTCTCT | This study | N/A |
| human *TSG6* Forward  GGAGTGTGGTGGCGTCTTTAC | This study | N/A |
| human *TSG6* Reverse  TGAGTCTAATGTGCCAGTAGCAGAT | This study | N/A |
| human *HO1* Forward  AGGGAAGCCCCCACTCAAC | This study | N/A |
| human *HO1* Reverse  ACTGTCGCCACCAGAAAGCT | This study | N/A |
| human *TGFB1* Forward  AGTTCAAGCAGAGTACACACAGCAT | This study | N/A |
| human *TGFB1* Reverse  AGAGCAACACGGGTTCAGGTA | This study | N/A |
| human *FGF2* Forward  AAGCGGCTGTACTGCAAAAAC | This study | N/A |
| human *FGF2* Reverse  TTGATGTGAGGGTCGCTCTTC | This study | N/A |
| human *iNOS* Forward  GGTGGAAGCGGTAACAAAGG | This study | N/A |
| human *iNOS* Reverse  TGCTTGGTGGCGAAGATGA | This study | N/A |
| human *S1PR1* Forward  TGTCAGCCTCCGTGTTCAGTCT | This study | N/A |
| human *S1PR1* Reverse  CCCGTTGTGGAGTTTCATTTTC | This study | N/A |
| mouse *Tnfα* Forward  CCCTCACACTCAGATCATCTTCT | Lim et al. 2020 | N/A |
| mouse *Tnfα* Reverse  GCTACGACGTGGGCTACAG | Lim et al. 2020 | N/A |
| mouse *Il1β* Forward  CTACAGGCTCCGAGATGAACAAC | This study | N/A |
| mouse *Il1β* Reverse  GTCCATTGAGGTGGAGAGCTTTC | This study | N/A |
| mouse *Il6* Forward  CAGTTGCCTTCTTGGGACTGAT | This study | N/A |
| mouse *Il6* Reverse  TTGGGAGTGGTATCCTCTGTGA | This study | N/A |
| mouse *Ccl2* Forward  GGCTCAGCCAGATGCAGTTAA | This study | N/A |
| mouse *Ccl2* Reverse  CCAGCCTACTCATTGGGATCA | This study | N/A |
| **Oligonucleotides (qChIP primers)** | | |
| human *OCT4* Forward  GCACTGAGGTCCTGGAGGGG | Kim et al. 2018 | N/A |
| human *OCT4* Reverse  GGGTGGAGGAGAGGGAGTG | Kim et al. 2018 | N/A |
| **Oligonucleotide (BSS)** | Kim et al. 2018 | N/A |
| human *OCT4* Inner Forward  AAGTTTTTGTGGGGGATTTGTAT | Kim et al. 2018 | N/A |
| human *OCT4* Outer Forward  GTTAGAGGTTAAGGTTAGTGGGTG | Kim et al. 2018 | N/A |
| human *OCT4* Inner/Outer Reverse  AAACCTTAAAAACTTAACCAAATCC | Kim et al. 2018 | N/A |
| human *H19*-DMR Outer Forward  AGGTGTTTTAGTTTTATGGATGATGG | Kim et al. 2018 | N/A |
| human *H19*-DMR Inner Forward  TCCTATAAATATCCTATTCCCAAATAACC | Kim et al. 2018 | N/A |
| human *H19*-DMR Inner/Outer Reverse  TGTATAGTATATGGGTATTTTTGGAGGTTT | Kim et al. 2018 | N/A |

**CAPTION FOR DATA S1 AND S2**

Dataset S1. Source data for quantification analyses

Dataset S2. GI index values for each plot

An Excel file containing Datasets S1 and S2 can be accessed on the journal website.

**SUPPLEMENTARY REFERENCES**

1. Mushahary D, Spittler A, Kasper C, Weber V, Charwat V. Isolation, cultivation, and characterization of human mesenchymal stem cells. *Cytometry Part A : the journal of the International Society for Analytical Cytology*. 2018;93:19-31.

2. Lim J, Lee S, Ju H, et al. Valproic acid enforces the priming effect of sphingosine-1 phosphate on human mesenchymal stem cells. *Int J Mol Med*. 2017;40:739-747.

3. Lee S, Lim J, Lee JH, et al. Ascorbic acid 2-glucoside stably promotes the primitiveness of embryonic and mesenchymal stem cells through ten-eleven translocation- and cAMP-responsive element-binding protein-1-dependent mechanisms. *Antioxid Redox Signal*. 2020;32:35-59.

4. Lim J, Heo J, Ju H, et al. Glutathione dynamics determine the therapeutic efficacy of mesenchymal stem cells for graft-versus-host disease via CREB1-NRF2 pathway. *Sci Adv*. 2020;6:eaba1334.

5. Bunnell BA, Flaat M, Gagliardi C, Patel B, Ripoll C. Adipose-derived stem cells: isolation, expansion and differentiation. *Methods (San Diego, Calif)*. 2008;45:115-20.

6. Kim Y, Jin HJ, Heo J, et al. Small hypoxia-primed mesenchymal stem cells attenuate graft-versus-host disease. *Leukemia*. 2018;32:2672-2684.

7. Jeong EM, Yoon JH, Lim J, et al. Real-time monitoring of glutathione in living cells reveals that high glutathione levels are required to maintain stem cell function. *Stem Cell Reports*. 2018;10:600-614.

8. Jin HJ, Lee HJ, Heo J, et al. Senescence-associated MCP-1 secretion Is dependent on a decline in BMI1 in human mesenchymal stromal cells. *Antioxid Redox Signal*. 2016;24:471-85.

9. Pittenger MF, Discher DE, Peault BM, Phinney DG, Hare JM, Caplan AI. Mesenchymal stem cell perspective: cell biology to clinical progress. *NPJ Regenerative medicine*. 2019;4:22.

10. Jang YK, Kim M, Lee YH, Oh W, Yang YS, Choi SJ. Optimization of the therapeutic efficacy of human umbilical cord blood-mesenchymal stromal cells in an NSG mouse xenograft model of graft-versus-host disease. *Cytotherapy*. 2014;16:298-308.

11. Kim DS, Jang IK, Lee MW, et al. Enhanced immunosuppressive properties of human mesenchymal stem cells primed by interferon-gamma. *EBioMedicine*. 2018;28:261-273.

12. Polchert D, Sobinsky J, Douglas G, et al. IFN-γ activation of mesenchymal stem cells for treatment and prevention of graft versus host disease. *Eur J Immunol*. 2008;38:1745-55.

13. Jeong EM, Shin JW, Lim J, et al. Monitoring glutathione dynamics and heterogeneity in living stem cells. *International journal of stem cells*. 2019;12:367-379.

14. Heo J, Lim J, Lee S, et al. Sirt1 regulates DNA methylation and differentiation potential of embryonic stem cells by antagonizing Dnmt3l. *Cell Rep*. 2017;18:1930-1945.

15. Heo J, Noh BJ, Lee S, et al. Phosphorylation of TFCP2L1 by CDK1 is required for stem cell pluripotency and bladder carcinogenesis. *EMBO Mol Med*. 2020;12:e10880.

16. Wakao S, Kitada M, Kuroda Y, et al. Multilineage-differentiating stress-enduring (Muse) cells are a primary source of induced pluripotent stem cells in human fibroblasts. *Proceedings of the National Academy of Sciences of the United States of America*. 2011;108:9875-80.

17. Wakao S, Akashi H, Kushida Y, Dezawa M. Muse cells, newly found non-tumorigenic pluripotent stem cells, reside in human mesenchymal tissues. *Pathology international*. 2014;64:1-9.

18. Kuroda Y, Wakao S, Kitada M, Murakami T, Nojima M, Dezawa M. Isolation, culture and evaluation of multilineage-differentiating stress-enduring (Muse) cells. *Nature protocols*. 2013;8:1391-415.

19. Kucia M, Reca R, Campbell FR, et al. A population of very small embryonic-like (VSEL) CXCR4(+)SSEA-1(+)Oct-4+ stem cells identified in adult bone marrow. *Leukemia*. 2006;20:857-69.

20. Kucia M, Wysoczynski M, Ratajczak J, Ratajczak MZ. Identification of very small embryonic like (VSEL) stem cells in bone marrow. *Cell and tissue research*. 2008;331:125-34.

21. Zuba-Surma EK, Klich I, Greco N, Laughlin MJ, Ratajczak J, Ratajczak MZ. Optimization of isolation and further characterization of umbilical-cord-blood-derived very small embryonic/ epiblast-like stem cells (VSELs). *European journal of haematology*. 2010;84:34-46.

22. Zuba-Surma EK, Kucia M, Wu W, et al. Very small embryonic-like stem cells are present in adult murine organs: ImageStream-based morphological analysis and distribution studies. *Cytometry Part A : the journal of the International Society for Analytical Cytology*. 2008;73A:1116-27.

23. Ratajczak MZ. Why are hematopoietic stem cells so 'sexy'? on a search for developmental explanation. *Leukemia*. 2017;31:1671-1677.

24. Shin DM, Liu R, Klich I, et al. Molecular signature of adult bone marrow-purified very small embryonic-like stem cells supports their developmental epiblast/germ line origin. *Leukemia*. 2010;24:1450-1461.

25. Shin DM, Zuba-Surma EK, Wu W, et al. Novel epigenetic mechanisms that control pluripotency and quiescence of adult bone marrow-derived Oct4(+) very small embryonic-like stem cells. *Leukemia*. 2009;23:2042-2051.

26. Wang X, Dai J. Concise review: isoforms of OCT4 contribute to the confusing diversity in stem cell biology. *Stem Cells*. 2010;28:885-93.

27. Tobin LM, Healy ME, English K, Mahon BP. Human mesenchymal stem cells suppress donor CD4(+) T cell proliferation and reduce pathology in a humanized mouse model of acute graft-versus-host disease. *Clin Exp Immunol*. 2013;172:333-48.

28. Im A, Hakim FT, Pavletic SZ. Novel targets in the treatment of chronic graft-versus-host disease. *Leukemia*. 2017;31:543-554.

29. Gao L, Zhang Y, Hu B, et al. Phase II multicenter, randomized, double-blind controlled study of efficacy and safety of umbilical cord-derived mesenchymal stromal cells in the prophylaxis of chronic graft-versus-host disease after HLA-haploidentical stem-cell transplantation. *Journal of clinical oncology : official journal of the American Society of Clinical Oncology*. 2016;34:2843-50.

30. Weng JY, Du X, Geng SX, et al. Mesenchymal stem cell as salvage treatment for refractory chronic GVHD. *Bone Marrow Transplant*. 2010;45:1732-40.

31. Phinney DG. Functional heterogeneity of mesenchymal stem cells: implications for cell therapy. *J Cell Biochem*. 2012;113:2806-12.

32. Phinney DG. Biochemical heterogeneity of mesenchymal stem cell populations: clues to their therapeutic efficacy. *Cell Cycle*. 2007;6:2884-9.

**UNCROPPED WESTERN BLOT RESULT**

**
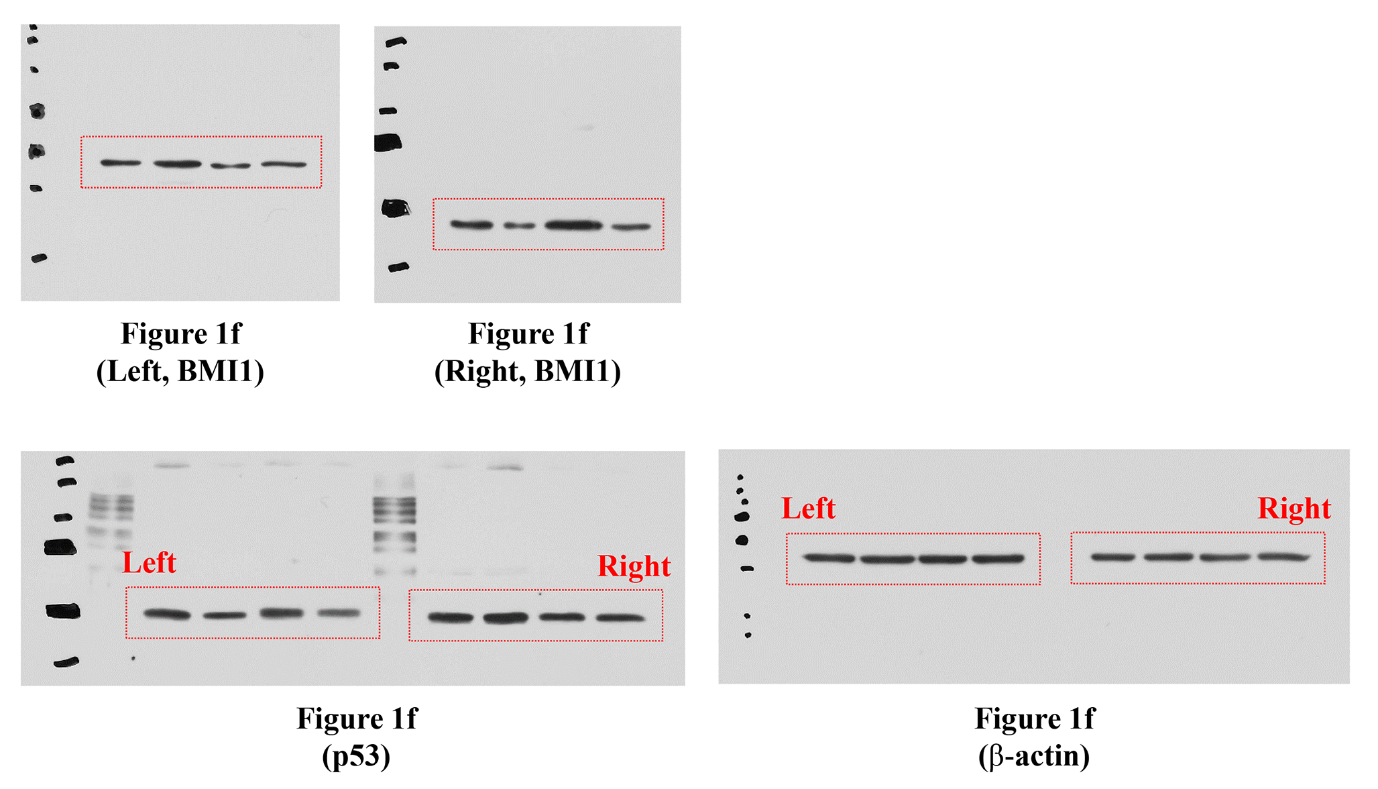
**
